# Infections and nutrient deficiencies during infancy predict impaired growth at 5 years: Findings from the MAL-ED study in Pakistan

**DOI:** 10.3389/fnut.2023.1104654

**Published:** 2023-02-17

**Authors:** Doris González-Fernández, Simon Cousens, Arjumand Rizvi, Imran Chauhadry, Sajid Bashir Soofi, Zulfiqar Ahmed Bhutta

**Affiliations:** ^1^SickKids Centre for Global Child Health, Toronto, ON, Canada; ^2^Department of Infectious Disease Epidemiology, Faculty of Epidemiology and Population Health, London School of Hygiene and Tropical Medicine, London, United Kingdom; ^3^Center of Excellence in Women and Child Health, The Aga Khan University, Karachi, Pakistan; ^4^Institute for Global Health and Development, The Aga Khan University, London, United Kingdom

**Keywords:** child growth, breastfeeding, complementary feeding, illness, environmental enteropathy, socio-demographic characteristics, intestinal infections, respiratory infection

## Abstract

**Background:**

Socio-economic, nutritional, and infectious factors have been associated with impaired infant growth, but how the presence of these factors during infancy affects growth around 5 years is not well understood.

**Methods:**

This secondary analysis of the MAL-ED cohort included 277 children from Pakistan for whom socio-demographic, breastfeeding, complementary foods, illness, nutritional biomarkers, stool pathogens and environmental enteropathy indicators between 0 and 11 months were recorded. We used linear regression models to analyze associations of these indicators with height-for-age (HAZ), weight-for-age (WAZ) and weight-for-height (WLZ) at 54–66 months (~5 years), and Poisson regression with robust standard errors to estimate risk ratios for stunting and underweight ~5 years, controlling for gender, first available weight, and income.

**Results:**

Among the 237 infants followed longitudinally and evaluated at about 5 years of age, exclusive breastfeeding was short (median = 14 days). Complementary feeding started before 6 months with rice, bread, noodles, or sugary foods. Roots, dairy products, fruits/vegetables, and animal-source foods were provided later than recommended (9–12 months). Anemia (70.9%), deficiencies in iron (22.0%), zinc (80.0%), vitamin A (53.4%) and iodine (13.3%) were common. Most infants (>90%) presented with diarrhea and respiratory infections in their first year. At ~5 years, low WAZ (mean-1.91 ± 0.06) and LAZ (−2.11 ± 0.06) resulted in high prevalence of stunting (55.5%) and underweight (44.4%) but a relatively low rate of wasting (5.5%). While 3.4% had concurrent stunting and wasting ~5 years, 37.8% of children had coexisting stunting and underweight. A higher income and receiving formula or dairy products during infancy were associated with a higher LAZ ~5 years, but infant’s history of hospitalizations and more respiratory infections were associated with lower LAZ and higher risk of stunting ~5 years. Infants’ intake of commercial baby foods and higher serum-transferrin receptors were associated with higher WAZ and lower risk of underweight ~5 years. Presence of *Campylobacter* and fecal neopterin >6.8 nmol/L in the first year were associated with increased risk of underweight ~5 years.

**Conclusion:**

Growth indicators ~5 years were associated with poverty, inappropriate complementary feeding, and infections during the first year of life, which supports the early start of public health interventions for preventing growth delay ~5 years.

## 1. Introduction

Despite some reduction, undernutrition continues to affect millions of children. According to the 2021 Global Nutrition Report, 149.2 million children under 5 suffer from stunting, 45.4 million are wasted (1). Also, and 1 in 2 children suffer from hidden hunger due to deficiencies in essential vitamins and nutrients (2). Undernutrition in the first 2 years of life has been linked with shorter adult height, lower educational achievement, reduced economic productivity and with smaller infants in the next generation (3). Linear growth is considered the best indicator of child well-being with stunting, defined as length/height < −2 SDs below the WHO child growth standard (4) indicative of past deprivation and predictive of future poverty (2). On the other hand, underweight (low weight-for-age), is known to increase the risk of viral, helminth and malaria infections in children (5). Wasting refers to children <-2SDs below the WHO standards weight-for-length/height median (6), reflecting a recent loss of weight from severe poor nutrient intake, illness or both (2). Children presenting both stunting and wasting have the highest risk of mortality, even higher than those with WLZ < -3 SD (7). Although recent reports show a decline in the global prevalence of stunting (from 32.5 to 21.9%) and wasting (from 10 to 7.3%) between 2000 and 2017, important disparities continue to be observed, south Asia presenting higher wasting at birth (19%) compared with African (8%) and Latin-American infants (2%) (7).

The origin of undernutrition is complex and multifactorial. The Etiology, Risk Factors and Interactions of Enteric Infections and Malnutrition and the Consequences for Child Health and Development (MAL-ED) study, followed children from eight LMIC (Bangladesh, India, Nepal, Pakistan, Brazil, Peru, South Africa and Tanzania), and showed, for example, that low energy and protein density of complementary foods and a high prevalence of enteropathogens in non-diarrheal stools were associated with reduced weight and length by age 24 months (8). MAL-ED studies have contributed to our understanding of the impact of environmental enteropathy (EE) on child growth. EE has been described as phenotypic intestinal alterations that affect the health status of the host, following repeated enteric infections even in the absence of diarrhea or acute gastrointestinal illnesses (9). The MAL-ED study used three fecal biomarkers of gut inflammation and immunity. Myeloperoxidase (ng/mL) is a marker of neutrophil activity in the intestinal mucosa, neopterin indicates T-helper cell 1 activity and alpha-1 antitrypsin is an indicator of protein loss and intestinal permeability (9). Indicators of EE were associated with reduced stature, weight, weight-for-height, and BMI at 5 years, but no associations were found between illness symptoms and size at 5 years (10).

Among countries in the MAL-ED study, Pakistan had a high prevalence of wasting (15%) and stunting (44%) according to the 2011 National Nutrition Survey (11). Pakistan also had the lowest mean WAZ (−1.4) and the highest prevalence of anemia in infants (88%) (12) compared with other cohorts in the study. Comparisons of home environment among MAL-ED sites found that children from Pakistan had the highest food insecurity scores (13), the shortest duration of exclusive breastfeeding (14) and the highest frequency of reported coughing (27%) (15). The prevalence of acute lower respiratory infections (ALRI) and ear pain were 6 and 7 times higher, respectively, compared with the next highest site (India) (15). In the MAL-ED cohort of Pakistan, persistent infection with *Giardia* was found to be associated with lower weight-for-age and length-for-age Z scores at 2 years (16). Although associations of other nutritional and infectious indicators, have been reported for other MAL-ED sites (8–10, 17, 18) similar analysis had not previously been performed for Pakistan after having detected field bias in length/height data collection.

Undernutrition is a major driver of health and economic consequences in Pakistan, and given stunting rates of 40%, wasting stagnating at around 15–17%, this is estimated to result in an approximate 3% loss of annual gross domestic product (19). Given that the highest incidence of stunting and wasting occurs in infancy (7), our objectives were to explore socio-demographic, nutrition, and infection factors in the first year of life and their association with anthropometry at 5 years in children from Pakistan.

## 2. Methods

**Figure 1:**
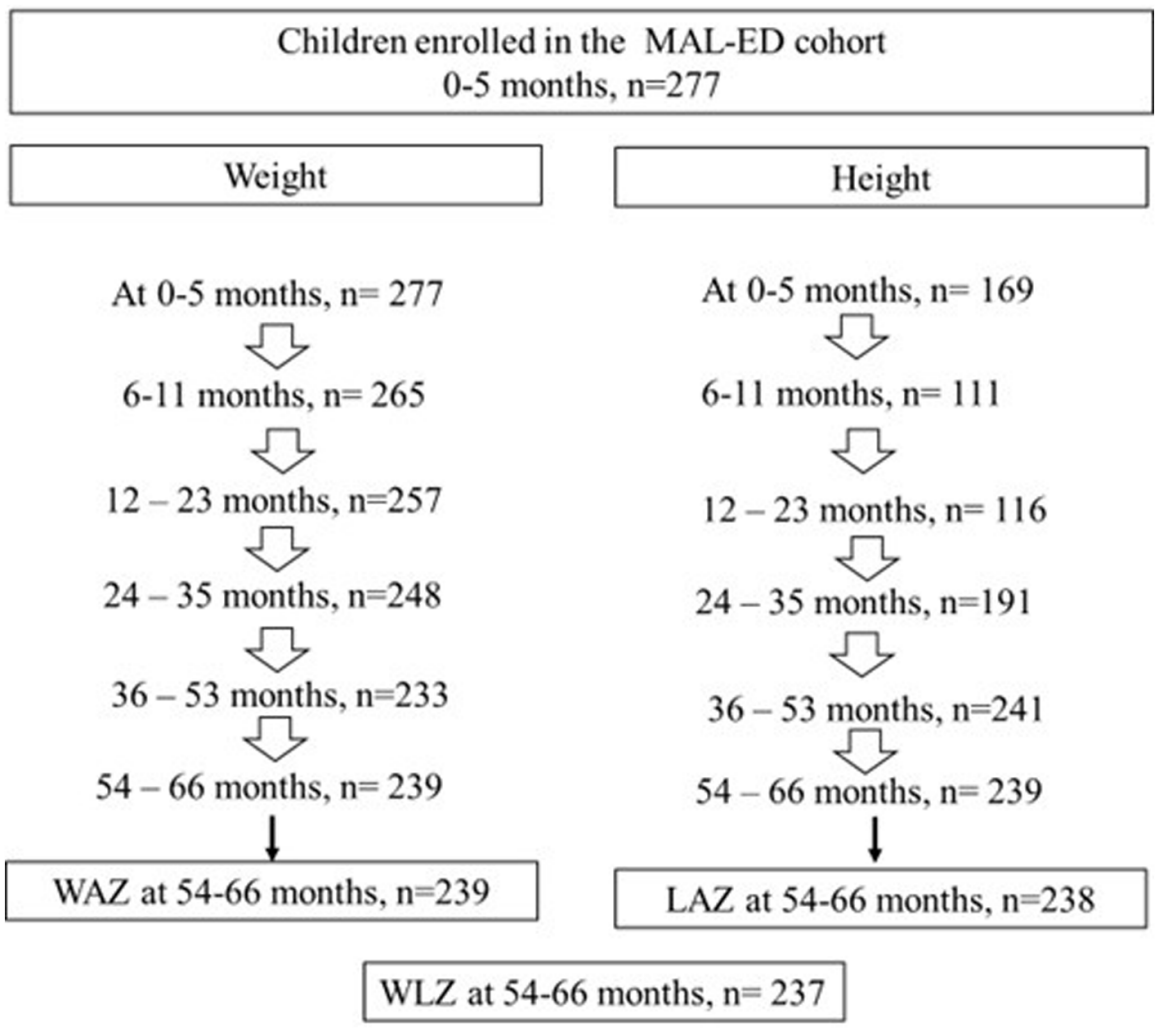
Sample size flow chart of children enrolled and followed between 0 and 5 years of age in the MAL-ED cohort in Pakistan.

This is a secondary analysis of data from the MAL-ED cohort of children from Pakistan, which recruited 277 healthy singleton newborns (≤17 days) from the Naushahro Feroze district in the Sindh province between January 2010 and February 2012 (11). Children were followed up to 66 months of age as shown in Figure 1, with a final available sample of 237 children at 54–66 months (86% of the original cohort). Length and weight were measured by trained personnel following standard procedures as previously described (8). A rigorous revisiting of length/height individual trajectories was performed, eliminating observations with possible bias during field data collection.

**Figure 2:**
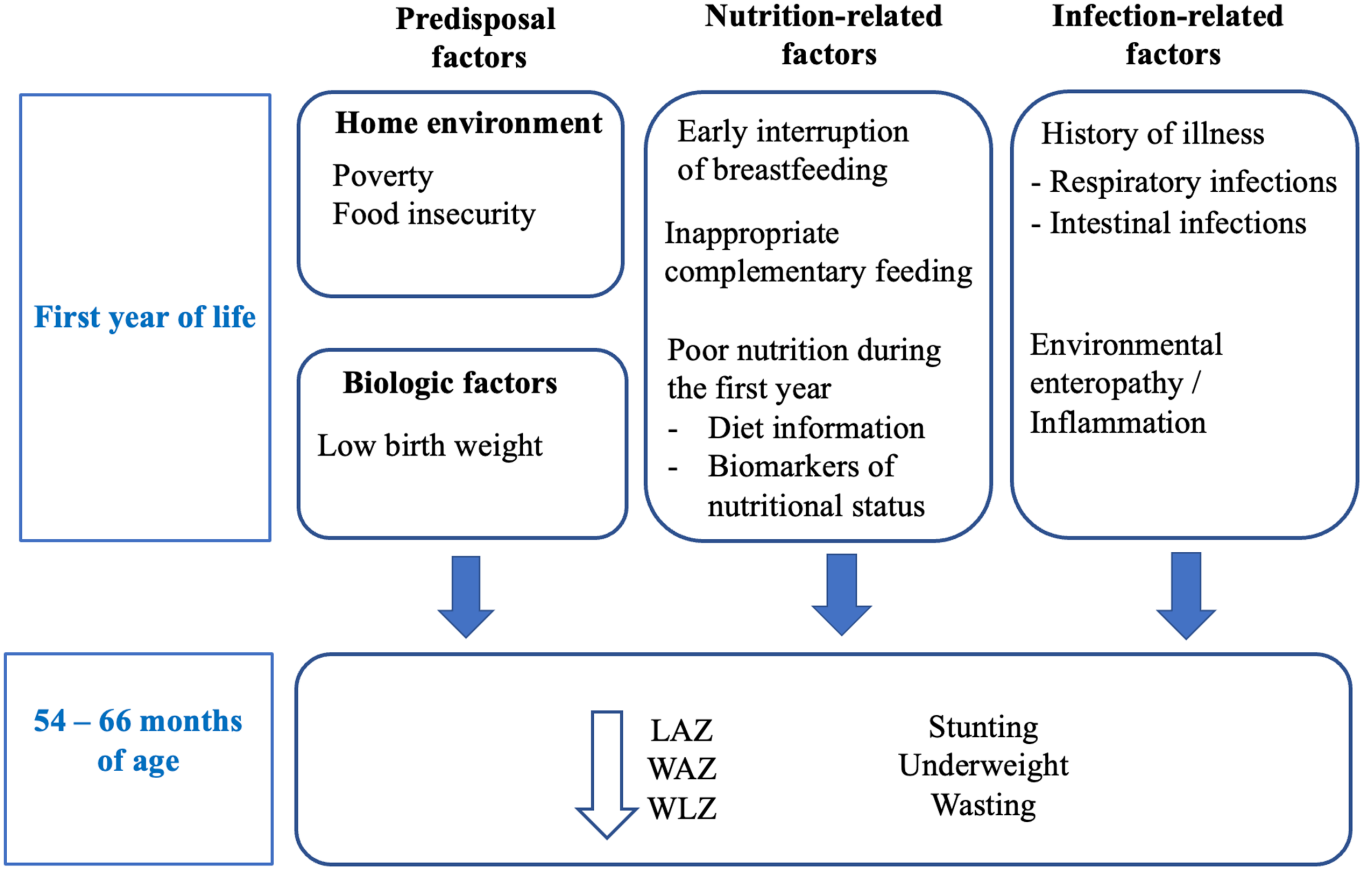
Conceptual framework: Known predictors of growth impairment include biological and environmental factors, nutrition-related factors, and infection-related factors. We hypothesize that adverse factors during the first year of life will be associated with indicators of growth at 54–66 months of age.

Our conceptual framework (Figure 2) identified possible early factors at 0–11 months of age that have known associations with child growth in Pakistan (20) or the MAL-ED cohort in other countries (8–10): (a) biological drivers (low birth weight), (b) adverse socio-demographic factors (c) inappropriate breastfeeding and weaning practices, (d) biomarkers of micronutrient deficiencies (iron indicators, vitamin A, zinc and iodine concentrations), (e) history of illness, and (f) biomarkers of infection and environmental enteropathy. These factors were grouped in clusters for further analyses.

### 2.1. Possible early determinants of impaired growth

- Biological drivers: Birth weight data was not available, but we controlled analyses for the first available weight, measured between 0 and 17 days of age. Early length data was missing for more than half of the sample (*n* = 111 infants) and was not used for analyses.
- Socio-demographic factors: number of assets (1–8 score including the sum of: bank account, chair, refrigerator, table, separate kitchen, TV, mattress, <2 people per room); food insecurity (mild, moderate, severe), material/kind of floor, roof walls and toilet (categorical variables); having sanitation at home (yes/no), household income (rupees/month) and number of people per room (continuous variables). Information on income, assets, maternal education, house characteristics (floor, roof and toilet material, having water/sanitation) has been previously reported (13). A nine-question survey adapted by the Food and Nutrition Technical Assistance (FANTA) project for use in low resource settings was used to assess food insecurity. The scale uses a four-week recall period and captures three dimensions of the access component of household food insecurity: anxiety and uncertainty about household food access, insufficient quality, and insufficient food intake and its physical consequences, that were used to categorize households as food access secure, and mildly, moderately and severely food access insecure (13). Socio-demographic variables did not differ between children with and without anthropometry information.
- Breastfeeding and weaning practices: Number of days receiving exclusive, predominant, partial or no breastfeeding (continuous variables), age of weaning, age at which non-milk fluids, milk and solid/semisolid foods were started (continuous variables), frequency (times/d) of breastfeeding, formula, milk, and meals between 0 and 5 months and between 6 and 8 months. Intake (yes/no) and age of starting different food groups: non-milk fluids, tea/coffee, formula, milk, grains, roots, legumes, fruits/vegetables, sweets, baby commercial foods, dairy products, animal-source foods. Groups of foods combinations (staples alone or in combination with legumes, food from animals, dairy products, fruits/vegetables) received between 6 and 11 months and the age of starting different foods were also explored. The age at which food groups (non-milk fluids, tea/coffee, milk, grains, legumes, dairy products, and animal-source foods) were introduced into child’s diet were previously described (21). More children with anthropometry data were fed with animal milk (74.5%) than children without anthropometry data (57.1%, *p* = 0.032). Children included in analyses had an earlier introduction of sweets in their diet [median: 5.9 months, range: (0.9–22.0) vs. 6.9 months (2.1–10.2), *p* = 0.009]. Other variables did not differ by having or not anthropometry data.
- Blood-urine biomarkers (in a subset of the population): alpha-1 glycoprotein (AGP, mg/dL), hemoglobin (g/dL), ferritin (μg/L), serum transferrin receptor (sTfR, mg/L), vitamin A (μg/dL), zinc (μmol/L), and urinary iodine (μmol/L) were assessed (21). Biomarkers did not differ in children who had and had not anthropometry data.
- Presence and severity of illness: mean days/month presenting fever (as reported by mothers, and temperature > 38°C as taken by interviewer), somnolence, low appetite, ALRI, diarrhea, ear pain/pulling, taking antibiotics; number of ALRI and diarrheal episodes, mean severity score of diarrheal episodes, mean proportion of days with diarrhea taking oral rehydration therapy (ORT). They were reported by mothers and by trained interviewers, together with the intake of oral rehydration solutions and antibiotics, and the need of hospitalization (22). Of note, diarrhea was defined as ≥3 loose stools in 24-h, or at least 1 loose stool with blood present, with episodes of diarrhea were separated by at least 3 diarrhea-free days (22). The severity of the episode was scored adding symptoms’ scales, taking in account duration (1: 2–4 days, 2: 5–7 days, 3: 8+ days), number of loose stools (1: <5, 2: 5–7, 3: 8+), days with vomit (1: 1 day, 2: 2 days, 3: 3+ days), dehydration (2: some dehydration, 3: severe dehydration), and presence of fever (0: no, 1: reported fever, 3: temperature > 37.5°C (23) Acute low respiratory infection (ALRI) was defined as the presence of cough and/or shortness of breath plus high respiratory rate (22). A higher proportion of children with data on anthropometry presented with diarrhea (99.2% vs. 84.2%, *p* < 0.0001), vomiting (96.2% vs. 81.6%, *p* < 0.0001), ALRI (95.8% vs. 81.6%, *p* = 0.001) and ear pain/pulling (82.8% vs. 60.5%, *p* = 0.001) than those without anthropometry data. Days per month presenting any illness did not differ between children with and without anthropometry information.
- Stool samples for microbiological analyses (24) and for fecal biomarkers of environmental enteropathy (25) were also collected:
  Pathogens: detection in stools at least once between 0 and 11 months of: Human astroviruses, human adenoviruses, *Norovirus*, *Aeromonas*, *Escherichia coli* (and pathotypes: enteroaggregative, enteroinvasive, enterotoxigenic, enteropathogenic, enterohemorrhagic and shiga-toxin producing *E. coli*), *Salmonella, Shigella, Vibrio, Campylobacter, Cryptosporidium, Entamoeba histolytica, and Giardia lamblia*. Other pathogens studied had too low positive observations to be included in analyses: *Endolimax nana* (*n* = 2), *Iodamoeba buetschlii* (*n* = 3), *Yersinia enterocolitica* (*n* = 1), *Balantidium coli*: (*n* = 0). A higher proportion of children with anthropometry data were found positive for *Campylobacter* (98.3%) compared with those without anthropometry data (85.7%, *p* = 0.002).
  Indicators of environmental enteropathy: myeloperoxidase (ng/mL), neopterin (nmol/L) and alpha-1 antitrypsin (mg/g). Given the lack of cut-offs indicating abnormal concentrations of fecal biomarkers in early infancy, if fecal biomarkers entered ≥500 repetitions, the Youden cut-point, which maximizes the sum of sensitivity and specificity (26) was calculated for the detection of the specific outcome of interest. Myeloperoxidase concentration was higher in children not included in analyses [median: 11.5 ng/mL, range (0.2–84.4 ng/mL)] compared with children with anthropometry information [2.2 (0.2–85.8) ng/mL]. Neither neopterin nor alpha-1 antitrypsin differed.

### 2.2. Statistical analyses

Outcome variables: Z-scores for weight-for-age (WAZ), height for age (HAZ), and weight for height (WHZ) at 5 years were calculated applying WHO reference data using STATA 16 (27) and analyzed as continuous variables, as well as their derivative binary variables underweight, stunting and coexistent underweight + stunting. The small number of wasted children at 5 years (5.5%) did not allow us to run models for this variable.

Spearman correlations among independent variables were explored for descriptive and modeling purposes. Descriptive statistics of variables for children having information on weight or height at 54–66 months were run (*n* = 239). Fisher’s exact, Chi^2^ or Kruskal-Wallis’s test were performed according to variable’s nature to identify if they differed between children with (*n* = 239) and without anthropometry data (*n* = 38).

#### 2.2.1. Variable pre-selection

Based on our conceptual framework, variables were aligned in groups of possible predictors. In order to select covariates that would best fit future modeling and discard noise variables (28), we used a bootstrapping procedure based on a backward stepwise algorithm with 1,000 repetitions (29), where groups of variables were tested avoiding the simultaneous inclusion of highly correlated variables, which were run separately.

#### 2.2.2. Univariate models

Only variables entering ≥500 repetitions were further analyzed in: (a) univariate models for binary (stunting, underweight, stunting + underweight) and (b) continuous (LAZ, WAZ, and WLZ) outcomes. We used generalized linear models (GLM) Poison and linear regression models, respectively. Dichotomous covariates with <10 events per variable were excluded (30). The univariate modeling process is shown in detail as Supplementary Material.

#### 2.2.3. Multivariable models

Variables with value of *p* is less than 0.05 in univariate models were further analyzed in multivariable regression models. We controlled all models for gender, first available weight and income (only socio-demographic variable entering ≥500 repetitions). GLM Poisson regression with robust standard errors were used to estimate risk ratios for binary outcomes, and multiple linear regression was used for continuous outcome variables. Backwards stepwise elimination (initially specifying value of *p* < 0.10, and decreasing value of *p* to <0.05 depending on the number of variables entering) were used to obtain final models including no more than 10 variables to avoid overfitting, using variables that are likely to be amenable to and influenced by interventions. Only final models are shown. Final models were tested for collinearity using a variance inflation factor < 10 and a condition number < 30 (31). Tests for covariate-dependent missingness (CDM) were conducted, allowing unequal variances between missing-value patterns (32).

## 3. Results

**Figure 3:**
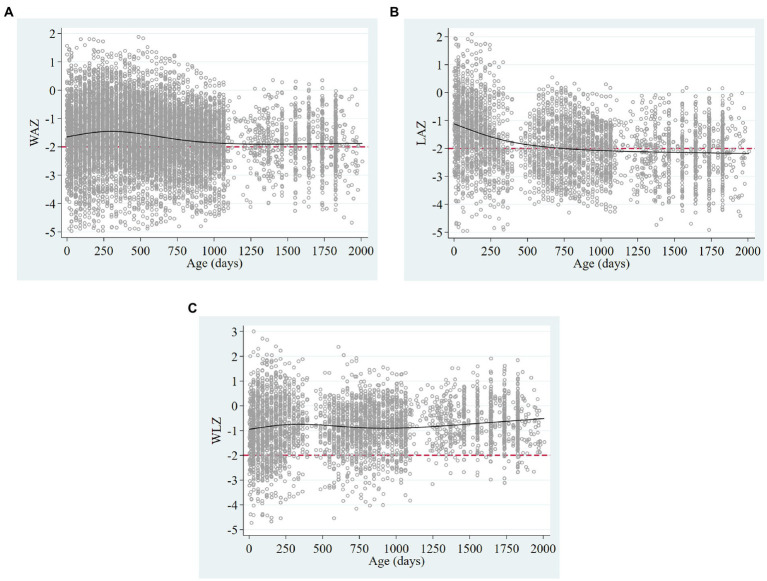
Scatter plots of **(A)** weight for age, **(B)** length/height, and **(C)** weight for length/height Z-scores, showing restricted cubic splines (solid black lines) and the cut-off of −2 standard deviations (dashed red lines) in children 0–5 years in the MAL-ED cohort of Pakistan.

**Figure 4:**
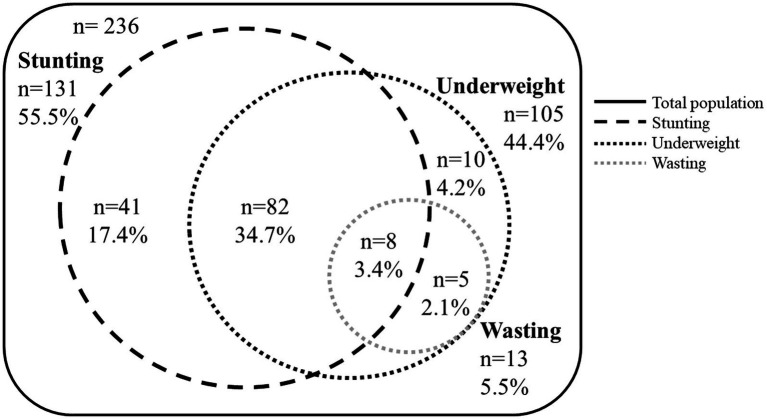
Venn diagram showing overlapping proportions of stunting, underweight, and wasting in children 54–66 months from Pakistan.

Characteristics of the population are described in Tables 1, 2; Figures 3, 4.

**Table 1:** Population characteristics.

Gender, girls	*n* = 120 (50.4%)
^1^Socio-demographic characteristics (*n* = 239)	Median (min-max) or prevalence, %
^2^Sum of assets	2 (0–8)
Maternal education, years	1 (0–16)
Non-educated mothers	49.8%
No. people living in the house	10 (3–29)
Floor material	
Earth, sand, clay, mud, dung	69.5%
Wood, ceramic, cement	30.5%
Roof material	
Thatch	17.2%
Metal, wood	12.1%
Brick, tiles	70.7%
Having sanitation at home	84.9%
Type of toilet	
No facility	19.6%
Pit latrine	8.4%
Flush to septic/sewer system	72.0%
^3^Monthly income (rupees × 10^3^), quantiles	12.5 (2.0–70.0)
<8 ×10^3^ rupees	23.4%
8–12.5	28.4%
>12.5–20	26.4%
>20–70	21.8%
^4^Food insecurity category	
No food insecurity	18.4%
Mild	9.2%
Moderate	64.0%
Severe	8.4%

^1^Socio-demographic information was collected at recruitment. ^2^Items included in the count of assets were mattress, chair, table, TV, refrigerator, bank account, having a separate kitchen and <2 people per room. ^3^One minimal wage at the time of the study was 8,000 rupees. ^4^Food insecurity questionnaires were administered at 0 and 6 months of age. Mean scores were used for analyses.

**Table 2:** Feeding practices and infections of Pakistani children during the first year of life.

^1^ A. Breastfeeding practices (*n* = 239)	Children receiving specific feeding (any duration) between 0 and 5 months		# Days between 0 and 5 months receiving specific feeding	
			Median (range)	IQR
Exclusive breastfeeding			14 (0–154)	8, 20
Predominantly breastfeeding			41 (0–164)	8, 89
Partial breastfeeding			99 (0–168)	41, 140
Frequency of feeding per day 0–5 months	*n*	%	Times/day between 0 and 5 months receiving specific feeding	
Breastmilk	239	100.0	16 (4–25)	14, 17
Formula	31	13.0	3 (1–16)	2, 4
Animal milk	178	74.5	2 (1–14)	1, 4
^2^B. Complementary feeding (*n* = 239)	Children receiving specific foods between 6 and 11 months (*n* = 258–277)		Age in months of complementary foods introduction	
	*n*	%	Median (range)	IQR
Non-milk fluids (e.g., water, tea/coffee, fruit juice, broth)	239	100.0	0.7 (0–10.1)	0.4, 2.0
Milk	153	64.3	1.0 (0–15.9)	0.5, 2.5
Formula	13	5.5	1.9 (0.9–7.0)	0.9, 3.0
Solid/semisolid foods	232	97.5	3.5 (0.03–10.0)	1.8, 4.8
Grains (e.g., rice, porridge, bread, noodles) (*n* = 232)	225	97.0	5.0 (0.8–10.9)	4.0, 6.0
Sugary foods (e.g., pastries, cakes, biscuits) (*n* = 232)	219	94.4	5.9 (0.9–22.0)	3.9, 6.9
Commercial baby foods (*n* = 232)	23	9.9	7.0 (4.0–8.1)	6.0, 7.9
Dairy products (e.g., cheese, yogurt) (*n* = 232)	10	4.3	9.0 (0.9–17.1)	8.9, 9.1
Roots (e.g., potatoes, yams, manioc) (*n* = 232)	80	34.5	9.1 (4.9–22.1)	7.9, 11.9
Fruits/vegetables (*n* = 232)	20	8.6	13.0 (4.0–59.5)	9.9, 17.0
Legumes (*n* = 232)	13	5.6	20.9 (3.8–60.1)	15.9, 27.9
Animal-source foods (*n* = 232)	14	6.0	22.0 (6.0–59.8)	13.2, 28.1
Age (months) stopping breastfeeding (*n* = 232)	230		19 (2–35)	14, 24
^3^C. Biomarkers (evaluated between 6 and 8 months)	# Samples	Deficiency, cut-off	Concentrations of blood/urine biomarkers	
		%	Median (range)	IQR
Hemoglobin (g/dL)	220	70.9% <11.0 g/dL	10.3 (7.0–14.5)	9.3, 11.1
Ferritin (μg/L)	185	22.0% <12 μg/L	22.5 (2.0–293.0)	14.0, 40.0
sTfR (mg/L)	216	1.4% >8.3 mg/L	2.9 (0.2–10.0)	1.7, 4.5
Vitamin A (μg/dL)	206	53.4% <20 μg/dL	19.3 (4.6–66.9)	14.4, 28.5
Zinc (μmol/L)	215	80.0% <10.7 μmol/L	8.9 (4.7–22.8)	7.8, 10.4
Urine iodine (μmol/L)	233	13.3% <100 μmol/L	240.4 (29.9–863.1)	143.2, 374.4
AGP (mg/dL)	215	36.7% >100 mg/dL	89 (1–223)	65.0, 115.0
^4^D. Reported illness information (*n* = 239)	Children presenting illness at least once between 0 and 11 months		Days per month having illness or number of hospitalizations between 0–11 months	
	n	%	Median (range)	IQR
Any illness	239	100	23 (5–30)	17, 26
Diarrhea	237	99.2	3 (0–25)	1.5, 5.6
Vomiting	230	96.2	1 (0–30)	0.4, 5.9
Acute low respiratory infection	229	95.8	1 (0–4)	0.2, 1.1
Ear pain/pulling	198	82.8	1 (0–26)	0.2, 3.9
Receiving antibiotics	238	99.6	5 (0–18)	3.2, 8.0
Hospitalized in the first year	13	5.4	In *n* = 13: 2 (1–8)	2, 4
^5^E. Fecal biomarkers, 0–11 months (*n* = 239)			Fecal concentration	
	*n*		Median (range)	IQR
Myeloperoxidase (ng/mL)	239		2.2 (0.2–85.8)	1.1, 7.4
Neopterin (nmol/L)	239		2.2 (0.02–56.9)	1.2, 4.9
Alpha-1-antitrypsin (μg/g)	239		4.7 (0.1–84.5)	1.8, 9.3
^6^F. Pathogens in stools during diarrheal episodes (0–11 months) (*n* = 239)	Children (+) for pathogens at least once between 0 and 5 months		Children (+) for pathogens at least once between 6 and 11 months	
	*n*	%	*n*	%
*Escherichia coli*	239	100	239	100
Enteroaggregative	214	89.5	216	90.4
Enteropathogenic	97	40.6	124	51.9
Enterotoxigenic	56	23.4	94	39.3
Shiga-toxin producing	10	4.2	8	3.3
Enterohemorrhagic	10	4.2	8	3.3
Enteroinvasive	1	0.4	5	2.1
*Campylobacter*	192	80.3	231	96.6
*Norovirus*	122	51.0	127	53.1
*Giardia*	104	43.5	176	73.6
*Aeromonas*	84	35.1	106	44.3
*Cryptosporidium*	71	29.7	78	32.6
Astroviruses	58	24.3	48	20.1
Adenoviruses	29	12.1	53	22.2
*Rotavirus*	32	13.4	33	13.8
*Vibrio*	18	7.5	20	8.4
*Shigella*	3	1.3	15	6.3
*Salmonella*	5	2.1	18	7.5
*Entamoeba histolytica*	4	1.7	8	3.3

^1^Daily breastfeeding information was available. Mean days per month children were fed exclusive, predominantly, partial or no breastfeeding were calculated for the first 6 months of life. ^2^Dietary information was recorded monthly during the first 8 months of life and food frequency questionnaires took place quarterly between 8 and 12 months of age. Compiled information was used to assess complementary feeding practices. ^3^An average of 3 blood/urine samples for nutritional biomarkers were taken between 6 and 8 months of age. Means of these three samples were used for calculating statistics. ^4^Daily illness information was recorded. Mean days per month presenting illness were calculated. ^5^Fecal biomarkers were available biweekly from 0 to 12 months of age. Mean concentrations during this period were calculated. ^6^Detection of pathogens in stools was performed monthly from 0 to 12 months of age.

### 3.1. Socio-demographic characteristics and food security

Characteristics of children’s families recruited in the study are shown in Table 1. Children came from families with low incomes, where 23.4% of them earned <8,000 rupees/month (equivalent to 100 USD), the minimal wage at the time of the study. Also, 81.5% lived with some degree of food insecurity. Overcrowding was common, with >3 people per room observed in 75% of homes. Although all households had water from tube well or borehole, 15% did not have sanitation. Among mothers, 50% had not received any education. Gender distribution of our sample showed similar proportions of boys and girls (50.4% girls).

### 3.2. Prevalence of stunting, underweight and wasting

First available weights (mean ± SD: 2.8 ± 0.5 kg between 0 and 17 days) found that 20.9% of children were below 2.5 kg. Among children with length available during the first month of age (*n* = 111), 39 (29.5%) were already stunted. Of note, most of these children continued to be stunted at 54–66 months (*n* = 25, 64.1%).

Figure 3 shows trajectories of WAZ, LAZ and WLZ of children from 0–5 years. Mean yearly lengths-for-age indicate that prevalence of stunting increased with age from 39.6% in the first year to 55.5% at 54–66 months. Similarly, underweight increased from 30% in the first year to 44.4% at 54–66 months. The presence of wasting (5.5% at 54–66 months) did not reflect the high proportions of underweight and stunting, as parallel and symmetric low weights and heights were observed in this population, but 37.8% had coexisting stunting and underweight. Overlapping proportions of stunting, underweight and wasting at 54–66 months are shown in Figure 4.

### 3.3. Breastfeeding and complementary feeding practices

Feeding practices are summarized in Table 2. Although all children received breastfeeding for some time between 0 and 5 months with a median frequency of 16 times/d, the median duration of exclusive breastfeeding was only 14 days. During their first month of age, most children (80.8%) received non-milk fluids (sugar water, thin soup or broth, fruit juice, tea, other fluids), animal milk/formula (29.2%) or both (25.5%). Between 0 and 5 months, most children received animal milk (74.5%, median frequency 2 times/d) or formula (13.0%, median frequency 3 times/d) starting at a median age of 1 month (Table 2A).

Complementary feeding with solid/semisolid foods started at a median age of 3.5 months, and was composed mainly of grains (rice, porridge, bread, noodles) and sugary foods (pastries, cakes, biscuits). After 6 months, children started receiving other dairy products (e.g., cheese, yogurt) and roots, whereas other nutritious foods such as legumes, fruits, and animal-source foods (animal flesh, eggs) were mostly introduced after 1 year of age (Table 2B).

### 3.4. Nutritional biomarkers

In the subsample of children (*n* = 186–220) with blood samples taken between 6 and 8 months of age, 89.0% had zinc deficiency, 71.3% had anemia, 53.7% had vitamin A deficiency, 22.1% had low ferritin but only 1.2% had elevated sTfR. Moreover, urine iodine was low in 13.2% of children. Finally, inflammation indicated by AGP >100 mg/dl was found in 38.7% (Table 2C). Of note, among children with anemia, 88.4% had also zinc deficiency, 57.4% had low vitamin A, 43.1% had elevated AGP, and 25.8% were iron deficient.

### 3.5. Illness information

On average, children spend 3 weeks/month presenting some type of illness, notably diarrhea, vomiting, respiratory infection, or ear pain/pulling, and nearly all of them received at least one cycle of antibiotics in the first month of life (Table 2D).

Microbiological stool sample analyses showed that the predominant pathogens present during diarrheal episodes were Enteroaggregative and Enteropathogenic *E. coli*, *Campylobacter* and *Norovirus*, which were isolated in more than half of children with diarrhea between 0 and 11 months (Table 2F).

#### 3.5.1. Fecal biomarkers

Concentrations of fecal biomarkers myeloperoxidase, neopterin and alpha-1-antitrypsin are shown in Table 2E. When assessing correlations between mean fecal biomarkers during the first year with indicators of infection/inflammation, neither mean myeloperoxidase nor neopterin concentrations correlated with serum AGP concentrations, but mean alpha-1-antitrypsin had a weak negative correlation with AGP (r_s_ = −0.16, *p* = 0.015). Fecal biomarkers did not correlate with the number of diarrheal episodes and did not differ by the presence/absence of intestinal pathogens in the first year. Higher mean concentrations of myeloperoxidase (r_s_ = −0.13, *p* = −0.029), neopterin (r_s_ = 0.17, *p* = 0.004) and alpha-1-antitrypsin (r_s_ = 0.32, *p* < 0.0001) were correlated with days/month presenting vomiting, and higher neopterin (r_s_ = 0.12, p-0.04) and alpha-1 antitrypsin (r_s_ = 0.19, *p* = 0.001) were correlated with more days/month presenting any illness during the first year of life. The cut-point of elevated neopterin for the detection of underweight using the Youden index was calculated at >6.8 nmol/L, and for the detection of underweight + stunting was >2.4 nmol/L.

### 3.6. Early predictors of impaired growth at 54–66 months of age

Univariate models for stunting, underweight and stunting + underweight are presented in Supplementary Tables S1–S3, respectively. Results of multivariate GLM-Poisson regression models are as follows:

#### 3.6.1. Stunting

Among variables selected using univariate models, only the average of ≥2 days per month presenting ALRI during the first year of life was associated with increased risk of stunting at 54–66 months (Table 3A). Variables with weak evidence of an association with increased risk of stunting at 54–66 months (entered the model with value of *p* >0.05) were being a girl, lower weight during the first years of life, lower income, and history of hospitalization in the first year.

**Table 3:** Multivariate logistic regression models for **(A)** stunting, **(B)** underweight, and **(C)** combined stunting + underweight compared with non-stunted/non-underweight children at 54–66 months.

(A) Model for stunting at 54–66 months	RR	95% CI	Value of *p*
Girls (*n* = 120)	1.26	0.99, 1.60	0.054
First available weight, kg	0.82	0.64, 1.05	0.118
Income (rupees × 10^3^, quantiles) base: 20.1–70 (*n* = 52)			
<8 (*n* = 56)	1.38	0.97, 1.96	0.074
8–12.5 (*n* = 67)	1.31	0.92, 1.85	0.130
12.6–20 (*n* = 63)	1.09	0.75, 1.58	0.645
Hospitalized at least once between 0 and 11 moths (*n* = 13)	1.32	0.98, 1.78	0.070
Mean days/month presenting ALRI between 6 and 11 months base: 0 (*n* = 119)			
1 (*n* = 94)	1.05	0.82, 1.36	0.679
2 (*n* = 16)	1.55	1.09, 2.20	0.015
3–4 (*n* = 9)	1.71	1.22, 2.41	0.002
Constant	0.50	0.20, 1.24	0.137
(B) Model for underweight at 54–66 months	RR	95% CI	value of p
Girls (*n* = 111)	1.05	0.81, 1.37	0.685
First available weight, kg	0.33	0.23, 0.46	<0.0001
Income (rupees × 10^3^, quantiles) base: 20.1–70 (*n* = 44)			
<8 (*n* = 51)	1.20	0.82, 1.77	0.347
8–12.5 (*n* = 62)	1.22	0.83, 1.80	0.314
12.6–20 (*n* = 59)	0.97	0.64, 1.47	0.897
*Campylobacter* (+) at least once between 0 and 5 months (*n* = 174)	1.78	1.17, 2.70	0.007
Received formula between 0 and 5 months (*n* = 25)	0.53	0.30, 0.96	0.036
Received commercial foods between 6 and 11 months (*n* = 23)	0.31	0.15, 0.62	0.001
sTfR (mg/L), quantiles base: 0.2–1.8 mg/L (*n* = 58)			
1.81–3.0 (*n* = 54)	0.96	0.67, 1.37	0.811
3.1–4.5 (*n* = 52)	1.01	0.73, 1.38	0.965
4.6–10.0 (*n* = 52)	0.55	0.35, 0.85	0.008
Neopterin 0–11 months >6.8 nmol/L (*n* = 21)	1.76	1.32, 2.35	<0.0001
Constant	5.99	1.98, 18.18	0.002
(C) Model for underweight + stunting at 54-66 months	RR	95% CI	value of p
Girls (*n* = 97)	1.24	0.92, 1.67	0.151
First available weight, kg	0.53	0.38, 0.73	<0.0001
Income (rupees x10^3^, quantiles) base: 20.1–70 (*n* = 49)			
<8 (*n* = 55)	2.08	1.25, 3.47	0.005
8–12.5 (*n* = 53)	1.77	1.05, 2.98	0.032
12.6–20 (*n* = 40)	1.34	0.77, 2.37	0.299
Hospitalized between 0 and 11 months (*n* = 11)	1.40	0.94, 2.10	0.101
Mean days/month presenting ALRI between 6 and 11 months base: 0 (*n* = 98)			
1 (*n* = 80)	1.07	0.77, 1.47	0.695
2 (*n* = 13)	1.41	0.85, 2.32	0.179
3–4 (*n* = 6)	1.63	1.08, 2.44	0.019
Neopterin 0–11 months >6.8 nmol/L (*n* = 23)	1.56	1.13, 2.26	0.009
Constant	0.99	0.32, 3.09	0.986

(A) Model *n* = 238, VIF = 1.02, condition number = 18.79. (B) Model *n* = 210, VIF = 1.07, condition number = 20.55. (C) Model *n* = 197, VIF = 1.02, condition number = 19.08. Little’s CDM test: *p* = 0.99.

#### 3.6.2. Underweight

The multivariable regression model for underweight at 54–66 months indicated that a higher weight in the first days of life, receiving formula in the first 6 months and receiving commercial baby foods between 6 and 11 months were associated with decreased risk of underweight at 54–66 months. Higher concentrations of sTfR (but below pathological concentrations, between 4.6 and 10 mg/l) were also associated with decreased risk of underweight at 54–66 months. Having *Campylobacter* infection in the first 6 months and having mean concentrations of neopterin >6.8 nmol/L in the first year of life were associated with increased risk of underweight at 54–66 months (Table 3B). Neither gender nor income appeared to be associated with underweight at 54–6 months.

#### 3.6.3. Underweight + stunting

When looking at early factors that might drive the presence of both stunting and underweight at 54–66 months compared with non-stunted/non-underweight children, having lower weight the first days of life, family income in the lower quantiles, more days/month presenting ALRI and neopterin >6.8 nmol/L in the first year (Youden’s cut-point for underweight) were associated with higher risk of underweight + stunting at 54–66 months (Table 3C). The Youden’s cut-point of neopterin >2.4 nmol/L for the detection of underweight + stunting at 54–66 months was not associated with this outcome when running the univariate or multivariate model. Gender was not associated with the presence of underweight + stunting at 54–66 months, and “hospitalized between 0 and 11 months” showed weak evidence of an association to increased risk of underweight + stunting at 54–66 months with a value of *p* = 0.105.

### 3.7. Early predictors of LAZ, WAZ, and WLZ at 54–66 months

Univariate models for LAZ, WAZ and WLZ are presented in Supplementary Tables S4–S6, respectively. Results of multivariate linear regression models are as follows (Table 4):

**Table 4:** Multivariate linear regression models for **(A)** LAZ, **(B)** WAZ, and **(C)** WLZ.

(A) Model for LAZ at 54-66 months	Coeff ± SE	95% CI	Value of p
Girls	−0.22 ± 0.12	−0.46, 0.02	0.076
First available weight, kg	0.53 ± 0.12	0.29, 0.78	<0.0001
Income (rupees x10^3^, quantiles) Base: 20.1–70 (*n* = 49)			
<8 (*n* = 55)	−0.43 ± 0.18	−0.79, −0.08	0.016
8–12.5 (*n* = 53)	−0.37 ± 0.17	−0.71, −0.03	0.031
12.6–20 (*n* = 40)	−0.16 ± 0.17	−0.50, 0.18	0.361
Received formula between 0 and 5 months (*n* = 31)	0.63 ± 0.18	0.27, 0.99	0.001
Received dairy products between 0 and 5 months (*n* = 19)	0.49 ± 0.21	0.08, 0.91	0.020
Hospitalized at least once between 0 and 11 months (*n* = 13)	−0.83 ± 0.28	−1.38, −0.28	0.003
Number of ALRI episodes between 0 and 11 months base: 0–1 (*n* = 47)			
2–4 (*n* = 124)	−0.37 ± 0.15	−0.67, −0.07	0.017
5–10 (*n* = 67)	−0.50 ± 0.18	−0.85, −0.15	0.005
Constant	−2.80 ± 0.47	−3.72, −1.88	<0.0001
(B) Model for WAZ at 54-66 months	Coeff. ± SE	95% CI	Value of *p*
Girls (*n* = 111)	−0.05 ± 0.11	−0.28, 0.16	0.610
First available weight, kg	0.69 ± 0.11	0.47, 0.92	<0.0001
Income (rupees × 10^3^, quantiles) base: 20.1–70 (*n* = 44)			
<8 (*n* = 51)	−0.12 ± 0.17	−0.45, 0.21	0.487
8–12.5 (*n* = 62)	−0.29 ± 0.16	−0.61, 0.03	0.078
12.6–20 (*n* = 59)	−0.05 ± 0.16	−0.37, 0.27	0.749
Receiving baby commercial foods between 6 and 11 months (*n* = 23)	0.69 ± 0.11	0.41, 1.12	<0.0001
Mean days/month presenting low appetite between 0 and 5 months base: 0 (*n* = 157)			
1 (*n* = 45)	−0.12 ± 0.15	−0.42, 0.17	0.408
2–8 (*n* = 14)	−0.40 ± 0.25	−0.88, 0.09	0.107
sTfR (mg/L), quantiles base: 0.2–1.8 mg/L (*n* = 58)			
1.81–3.0 (*n* = 54)	0.22 ± 0.16	−0.10, 0.55	0.171
3.1–4.5 (*n* = 52)	0.10 ± 0.17	−0.24, 0.45	0.545
4.6–10.0 (*n* = 52)	0.59 ± 0.18	0.24, 0.94	0.001
Constant	−3.97 ± 0.43	−4.81, −3.13	<0.0001
(C) Model for WLZ at 54–66 months	Coeff. ± SE	95% CI	Value of *p*
Girls (*n* = 111)	0.12 ± 0.11	−0.09, 0.33	0.272
First available weight, kg	0.46 ± 0.11	0.23, 0.68	<0.0001
Income (rupees × 10^3^, quantiles) base: 20.1–70 (*n* = 44)			
<8 (*n* = 50)	0.20 ± 0.17	−0.13, 0.53	0.235
8–12.5 (*n* = 61)	0.07 ± 0.16	−0.25, 0.38	0.669
12.6–20 (*n* = 59)	0.12 ± 0.16	−0.20, 0.44	0.460
Age starting milk base: 6–20 months (*n* = 24)			
<1 month (*n* = 105)	−0.28 ± 0.18	−0.64, 0.07	0.116
1–2 months (*n* = 53)	−0.44 ± 0.19	−0.83, −0.06	0.024
3–5 months (*n* = 28)	−0.01 ± 0.22	−0.42, 0.44	0.950
Received other fluids between 6 and 11 months (*n* = 153)	0.25 ± 0.12	0.004, 0.49	0.046
Received baby commercial foods between 6 and 11 months (*n* = 22)	0.67 ± 0.18	0.32, 1.03	<0.0001
Norovirus (+) at least once between 0 and 11 months (*n* = 153)	−0.19 ± 0.13	−0.44, −0.05	0.127
(C) Model for WLZ at 54–66 months	Coeff. ± SE	95% CI	Value of *p*
sTfR (mg/L), quantiles base: 0.2–1.8 mg/L (*n* = 57)			
1.81–3.0 (*n* = 54)	0.31 ± 0.15	0.01, 0.61	0.043
3.1–4.5 (*n* = 51)	0.04 ± 0.15	−0.26, 0.34	0.786
4.6–10.0 (*n* = 52)	0.58 ± 0.15	0.27, 0.88	<0.0001
Constant	−2.39 ± 0.46	−3.390-1,47	<0.0001

(A) Model *n* = 207, *p* < 0.0001, adj. R^2^ = 0.24, VIF = 1.07, condition number = 20.85. A preliminary backwards stepwise regression models specifying a *p* < 0.10, took out “receiving commercial products between 0 and 5 months” (*p* = 0.243) and “Mean days/month presenting vomit between 0 and 5 months.” The variable “Received > =1 portion/d of protein between 8 and 11 months (*n* = 12)” added heteroscedasticity to the model and was taken out. (B) Model *n* = 210, *p* < 0.0001, adj. R^2^ = 0.266, VIF = 1.15, condition number = 21.40. A preliminary backwards stepwise regression model specifying a *p* < 0.10, took out the variables “Taking ≥ 1 portion/d of animal-source foods between 8 and 11 months” and “Received ≥ 1 portion/d of fruit between 8 and 11 months.” (C) Model *n* = 194, *p* < 0.0001, adj. R^2^ = 0.22, VIF = 1.04, condition number = 24.11. A preliminary backwards stepwise regression models specifying a *p* < 0.05, adjusting for gender, income, first weight and hospitalizations in the first 5 months, took out the variables “Received any quantity of animal-source foods/d between 8 and 11 months,” “Days presenting low appetite between 0 and 5 months,” “Mean days/month presenting ALRI between 0 and 5 months.” Little’s CDM test: *p* = 0.99.

#### 3.7.1. LAZ

Increases in child length at 54–66 months were associated with a greater weight in the first days of life, higher family income, receiving formula and dairy products in the first 6 months of life, while lower child length was associated with having been hospitalized and the number of ALRI episodes during the first year of life. There was only weak evidence of an association between gender and LAZ (*p* = 0.076) (Table 4A).

#### 3.7.2. WAZ

Higher weight at 54–66 months was associated with greater weight during the first days of life, having received baby commercial foods after 6 months and a higher sTfR (but below abnormal values, between 4.6 and 10 mg/l) (Table 4B). Other variables that entered ≥500 repetitions but only showed weak evidence of an association with WAZ were “gender,” “income,” and “mean days/month having low appetite in the first 5 months.”

#### 3.7.3. WLZ

A higher weight-for-length was associated with greater weight during the first days of life, and with indicators of food availability during the complementary-food period. A too-early start of non-breast milk (1–2 months of age) was associated with lower WLZ at 54–66 months, but those children receiving baby commercial foods between 6 and 11 months and fluids such as sugar water, thin soup or broth or carbonated drinks between 0 and 11 months had higher WLZ at 54–66 months. sTfR in its highest quartile (4.6–10 mg/L) at 6–8 months was also associated with higher WLZ at 54–66 months. Neither gender nor income nor infection-related variables were associated with WLZ at 54–66 months (Table 4C).

## 4. Discussion

This study explored early predictors of undernutrition at 5 years among socio-demographic, breastfeeding and complementary feeding practices, illness, intestinal pathogens and indicators of environmental enteropathy during the first year of age in children from Pakistan. Chronic undernutrition was highly prevalent, evidenced by high rates of stunting and underweight (but not wasting) at 5 years. The low adherence to exclusive breastfeeding, combined with an early start of weaning consisting of non-milk fluids (sugar water, thin soup or broth, fruit juice, tea), and “empty-caloric” foods added to frequent and persisting infections, characterized the infancy of this cohort. Main findings that distinguished this cohort from other MAL-ED studied countries (10) included that infection during infancy, mainly lower respiratory infections, emerged as a strong predictor of LAZ at 5 years, whereas early infant intestinal colonization by *Campylobacter*, and one indicator of environmental enteropathy (neopterin >6.8 nmol/L) were associated with underweight at 5 years. Among nutritional indicators, initiation of complementary feeding with nutrient-dense foods was associated with higher WAZ and WLZ. Surprisingly, lower sTfR in the presence of anemia and iron deficiency during infancy was a predictor for impaired weight at 5 years, probably indicating a blunted erythropoietic response and early protein-calorie malnutrition, which may help to explain this unexpected association.

We acknowledge our limitation of a reduced sample size for children with accurate data on length/height between 0 and 35 months, but the rigorous data cleaning makes us confident of the accuracy of reported results. We lacked information on size at birth for many children, a possible parameter of importance for determining size at 5 years, and the lower sample size of children with blood biomarkers during the first year might have limited the power to find possible associations of specific nutrient deficiencies or systemic inflammation with growth at 5 years. Although the number of wasted children at 5 years did not allow to run analyses for this binary variable, the continuous WLZ was explored as continuous variable. Moreover, the richness of information gathered by the MAL-ED investigators allowed to elucidate important targets for future interventions to reduce child impairment in this population.

The prevalence of stunting (55.5%), underweight (44.4%) and wasting (5.5%) found in the MAL-ED cohort of children at 54–66 months from the Sindh province in 2015 was consistent with the prevalence reported by the Pakistan Demographic and Health Survey 2012–2013, where 45.6% stunting, 30.0% underweight and 4.8% wasting in children 48–59 months were reported (33). Although the use of stunting as an indicator of undernutrition has been debated (34), in particular the cut-off of −2 LAZ as definition of “chronic malnutrition” (35), it is clear that stunting reflects adverse growth conditions starting *in utero* and continuing during infancy, involving not only nutritional factors (36), but also the lack of appropriateness of children’s environment (37). Our results in this population with mean LAZ and WAZ <0, indicate that children are not achieving their growth potential (35), where 37.8% had coexistent underweight and stunting. Moreover, deficiencies in micronutrients that are essential for growth and development were extremely high in this population, in particular anemia (71.3%) and zinc (89.0%) deficiencies.

The importance of coexisting wasting and stunting has been emphasized as an indicator of children with higher risk of mortality (38, 39). Despite the low prevalence of wasting in our study, our results showed that predictors of WLZ were similar to those of WAZ. We also showed a high prevalence of concurrent low WAZ and LAZ. Our results add to the literature information on predictors of concurrent underweight and stunting, where most predictors of the coexistent condition overlap with those of stunting, suggesting a major contribution of illnesses (history of hospitalization and ALRI) and inflammation (elevated neopterin) over nutritional indicators as possible drivers of a more worrisome condition. These findings support the need of addressing infections together with nutritional interventions in the presence of coexisting underweight and stunting.

Among the multiple factors studied during the first year of life, only few remained as predictors of undernutrition at 5 years after a strict bootstrapping-selecting procedure. Other studies have shown lower income as a main determinant of undernutrition (19), but in our study lower LAZ was the only indicator associated with lower income. In contrast with other MAL-ED sites that did not find an association between illness and growth at 5 years (10), we found that history of hospitalization and more days/month presenting ALRI were associated with lower LAZ, and ALRI infections increased the risk of stunting at 5 years, in agreement with current knowledge of undernutrition and inflammation as common pathways for reduced linear growth in children (36).

The WHO recommends that infants initiate breastfeeding within the first hour of birth, and continue exclusively breastfeeding for the first 6 months of life (40). This is far from the case of the Pakistan MAL-ED cohort, where exclusive breastfeeding was provided with a median duration of only 2 weeks. Analyses showed that providing formula between 0 and 5 months was associated with reduced risk of underweight, and increased WAZ and LAZ. Of note, early start of complementary feeding was a common practice, where mothers provided mainly clear fluids or animal milk, but only 6% of children received formula, and those children came from families with higher incomes. Our findings highlight the importance of reinforcing the message of exclusive breastfeeding during the first 6 months in these communities, which would overcome the lack of resources to afford appropriate complementary foods at that early age. Providing formula has been suggested as a therapeutic approach to correct weight deficits in developing settings (41), but for populations like the MAL-ED cohort, such approach would not be neither financially nor context appropriate. On the other hand, a recent systematic review found that bovine/cow milk supplementation had unfavorable effects on infant morbidity and mortality (42), which aligns with our finding of earlier start of milk compared with starting milk after 6 months of age associated with lower WLZ. Also subject of controversy, commercial infant foods have shown a contribution of 70% of recommended nutrient intakes in children 13–23 months in Ghana (43), which suggest that our finding of better WAZ and WLZ in children who had consumed commercially available infant foods may be related to an improvement in their micronutrient status, but this would require further study.

It was intriguing to find that higher sTfR in the first year, at concentrations below the cut-off for iron deficiency, was associated with higher WAZ and decreased risk of underweight at 5 years. Of note, no other iron status indicators were associated with child growth indicators at 5 years. It is known that transferrin, the acute-phase reactant protein that transports iron, has been used as well as a marker of nutritional status, given that it parallels prealbumin concentrations during nutritional interventions and is decreased in severe malnutrition (44). Moreover, the total mass of sTfR depends on the number of erythroid precursors in the bone marrow (45), and that those are in turn decreased in protein-energy malnutrition (44). Our findings suggest that a deprived erythropoiesis during infancy expressed as low concentrations of sTfR despite iron deficiency and anemia are reflecting protein-energy malnutrition. Our findings mimic those of other MAL-ED sites, where higher sTfR were associated with higher WAZ (10).

The association between ALRI and impaired growth has been previously documented. Stunting increased the risk of ALRI in infants from Turkey (46) and in children under 5 in Sri Lanka (47), and acute respiratory infection was the major predictor of underweight in children under 5 from India (48). To our knowledge, ours is the first study reporting the association of ALRI in the first year of life with impaired growth at 5 years. Whereas much attention has been paid to diarrheal disease as a major cause of mortality in developing countries, ALRI is also an important cause of morbidity and mortality, which are proportional to socio-demographic conditions (49). Both viruses and bacteria share the etiology of ALRI in children, but the severity of bacterial ALRI largely exceeds that of viral ALRI. However, by the time of the study, Pakistan National data shows that 46.2% of children were not fully immunized (33). Vaccines against bacteria and viruses implicated in morbidity and mortality due to ALRI such as measles, diphtheria, pertussis, Haemophilus influenzae b, pneumococcus and influenza have the potential to reduce the burden of the disease (50), and therefore, could help in reducing growth failure in this particular setting.

A consistent association between intestinal *Campylobacter* infection and lower LAZ together with increased intestinal and systemic inflammation was found across other MAL-ED sites (51). Our findings extend this association from the earliest age of infection at 0–5 months to low WAZ at 5 years. The association of *Campylobacter* with malnutrition indicated by WAZ has been previously reported in children 6–23 months from Bangladesh (52). Given that host factors such as shifts in diet and microbiota can modify colonization resistance to the infection, *Campylobacter* has been proposed as a biomarker of enteropathy and is a potential target for interventions intending to improve child growth in developing countries (53).

The MAL-ED cohorts from Nepal and Bangladesh found an association of myeloperoxidase between 3 and 6 months with growth velocity from 9 to 24 months (54) and between myeloperoxidase between 3 and 21 months and successive 3-month change in LAZ (55) respectively, but did not find an association of neopterin or alpha-1 antitrypsin with linear growth. In the present study, we only found an association between higher neopterin at concentrations ≥6.8 nmol/L and increased risk of underweight at 5 years, but no association was found between any of the three EE indicators with LAZ or stunting. A higher fecal neopterin in children hospitalized with severe acute malnutrition compared with controls was found in a study from Uganda (56), and given that neopterin in our study was positively correlated with the number of days presenting all-cause illness in the first year, it is possible that a high fecal neopterin might be reflecting systemic inflammation to some extent. At this regard, there is strong evidence that the association between fecal biomarkers and impaired child growth is mediated through systemic inflammation (9). Therefore, neopterin appeared in our study as the earliest fecal biomarker that predicted undernutrition at 5 years.

## 5. Conclusion

Chronic malnutrition was highly prevalent in children from Pakistan, and our study evidenced associations with early inappropriate complementary feeding practices and illnesses, particularly respiratory infections, with lower growth indicators. Analyses suggested that the pathway for these associations could be early malnutrition indicated by low sTfR in the presence of iron deficiency and anemia, and inflammation indicated by higher fecal neopterin, which was in turn correlated with overall illness. Taken together, our findings suggest that reinforcing exclusive breastfeeding, facilitating the intake of nutritious foods after 6 months of age and preventing respiratory infections might help reducing the prevalence of child growth impairment in the Pakistani population.

## Data availability statement

The Mal-ED data sets are centrally available upon request from the MAL-ED data repository at the University of Pennsylvania. The analytical codes used for this analysis can be requested from the study authors.

## Ethics statement

The studies involving human participants were reviewed and approved by Ethics Review Committee Aga Khan University and the Foundation for the National Institutes of Health. Written informed consent to participate in this study was provided by the participants’ legal guardian/next of kin.

## Author contributions

ZB is the principal investigator for the Pakistan MalED cohort. For this analysis DG-F, SC and ZB jointly developed the analytical design, the conceptual framework and wrote the manuscript. DG-F and SC performed statistical analysis. AR, IC, SS, and the MAL-ED Pakistan investigators coordinated field and laboratory analyses, read, and approved the final manuscript. All authors contributed to the article and approved the submitted version.

## Funding

This study was supported by the Bill and Melinda Gates Foundation and by the Microbiome, Infections, and Childhood Growth and Development Fellowship Program of the Centre for Global Child Health, Toronto.

## Conflict of interest

The authors declare that the research was conducted in the absence of any commercial or financial relationships that could be construed as a potential conflict of interest.

## Publisher’s note

All claims expressed in this article are solely those of the authors and do not necessarily represent those of their affiliated organizations, or those of the publisher, the editors and the reviewers. Any product that may be evaluated in this article, or claim that may be made by its manufacturer, is not guaranteed or endorsed by the publisher.
