# Supplementary material for: Infections and nutrient deficiencies during infancy predict impaired growth at 5 years: Findings from the MAL-ED study in Pakistan
Source: Front Nutr. 2023 Feb 17;10:1104654. doi: 10.3389/fnut.2023.1104654 (PMC9982131; doi:10.3389/fnut.2023.1104654)
Supplement: Supplementary file 1 [file Data_Sheet_1.PDF]

Supplementary Material

Description of modeling process

Models exploring possible predictors of anthropometry at 5 years among socio-demographic characteristics, breastfeeding practices, diet, illness, intestinal pathogens and blood, urine, and fecal biomarkers.

Table S1. Univariate models for stunting at 54-66 months (n=238)

Controlling variables	Crude RR (95% CI)	p-value
Girls	1.17 (0.93, 1.47)	0.176
First available weight, kg	0.80 (0.62, 1.03)	0.079
Income (rupees x10^3, quantiles)		
Base: 20.1-70 (n=52)		
<8 (n=56)	1.44 (1.01, 2.04)	0.043
8-12.5 (n=67)	1.30 (0.91, 1.86)	0.145
12.6-20 (n=63)	1.13 (0.77, 1.65)	0.521
Constant	0.66 (0.28, 1.55)	0.343

Variable group	Variables entering ≥500 bootstrap repetitions	RR (95% CI)	Unadjusted p-value	RR (95% CI)	Adjusted for gender, first weight and income p-value
Socio-demographic	Number of assets				
	Base: 0 (n=44)				
	0-3 (n=105)	0.90 (0.67, 1.20)	0.480	0.97 (0.72, 1.30)	0.845
	4-8 (n=89)	0.84 (0.62, 1.15)	0.277	0.99 (0.68, 1.44)	0.961
Breastfeeding	Mean frequency of breastfeeding /day between 8-11 months				
	Base: ≤6 times (recommended, n=37)				
	7-11 (median) (n=98)	1.25 (0.83, 1.89)	0.288	1.21 (0.79, 1.84)	0.380
	12-18 (n=103)	1.39 (0.93, 2.08)	0.107	1.34 (0.89, 2.04)	0.160
	Mean times/d receiving milk between 0-5 months				
	Base: 1 (n=71)				
	2 (n=61)	0.95 (0.72, 1.26)	0.731	0.97 (0.74, 1.27)	0.835
	3-4 (n-32)	0.65 (0.41, 1.04)	0.071	0.65 (0.41, 1.03)	0.067
	5-11 (n=13)	0.74 (0.40, 1.38)	0.349	0.72 (0.38, 1.35)	0.309
Diet diversity	Age (months) when started roots				
	Base: ≥6 months (n=69)				
	0-5 months (n=14)	1.00, 1.82	0.050	1.29 (0.95, 1.74)	0.099
	Haven't received roots at 8 months (n=155)	1.07, 2.52	0.023	1.61 (1.07, 2.43)	0.022
Mean dietary intakes/day 0-8 months	Mean tea/coffee intake/d (cups) between 8-11 months				
	Base: 0 (n=62)				
	1 cup/d (n=112)	1.39 (1.01, 1.92)	0.042	1.39 (1.01, 1.90)	0.042
	2 cups (n=64)	1.29 (0.90, 1.85)	0.160	1.30 (0.92, 1.86)	0.138
	Receiving fruits at least once/d between 8-11 months (n=45)	0.72 (0.50, 1.05)	0.087	0.73 (0.50, 1.05)	0.092

Dietary intakes (yes/no) per period	Received formula between 0-5 months (n=31)	0.67 (0.42, 1.06)	0.091	0.68 (0.43 ,1.06)	0.091
	Received dairy products between 0-5 months (n=19)	0.54 (0.27, 1.06)	0.073	0.53 (0.28, 1.02)	0.057
	Received staples between 0-5 months (n=178)	1.41 (0.90, 2.22)	0.134	1.44 (0.93, 2.23)	0.103
	Received breastfeeding between 6-11 months (n=223)	1.99 (0.86, 4.61)	0.107	1.97 (0.85, 4.56)	0.112
	Received roots between 6-11 months (n=80)	0.79 (0.60, 1.03)	0.078	0.82 (0.63, 1.07)	0.142
	Received commercial foods between 6-11 months (n=23)	0.60 (0.34, 1.06)	0.078	0.59 (0.34, 1.02)	0.061
Illness	Hospitalized at least once between 0-11 moths (n=13)	1.43 (1.04, 1.97)	0.029	1.42 (1.06, 1.91)	0.020
	Mean days/month presenting ALRI between 0-11 months Base: 0 (n=111)				
	1 (n=90)	1.04 (0.80, 1.35)	0.780	1.04 (0.80, 1.35)	0.778
	2 (n=27)	1.37 (1.01, 1.86)	0.043	1.42 (1.04, 1.93)	0.026
	3-4 (n=10)	1.36 (0.87, 2.13)	0.173	1.43 (0.90, 2.27)	0.133
	Mean days/month presenting ALRI between 6-11 months				
	Base: 0 (n=119)				
	1 (n=94)	1.04 (0.80, 1.34)	0.779	1.05 (0.81, 1.36)	0.696
	2 (n=16)	1.46 (1.05, 2.04)	0.025	1.55 (1.08, 2.22)	0.017
	3-4 (n=9)	1.73 (1.30, 2.32)	<0.0001	1.78 (1.30, 2.45)	<0.0001
	Number of ALRI episodes between 6-11 months				
	Base: 0-1 (n=129)				
	2 (n=51)	1.18 (0.88, 1.58)	0.248	1.17 (0.88, 1.55)	0.287
	3 (n=31)	1.136 (1.01, 1.84)	0.041	1.37 (1.01, 1.84)	0.040
	4-6 (n=27)	1.19 (0.83, 1.71)	0.331	1.28 (0.88, 1.87)	0.189
	Number of diarrheal episodes between 6-11 months				
	Base: 0-1 (n=67)				
	2-3 (n=70)	0.91 (0.69, 1.20)	0.509	0.90 (0.6, 1.18)	0.448
	4-5 (n=52)	0.80 (0.57, 1.11)	0.178	0.79 (0.57, 1.11)	0.178
	5-7 (n=31)	0.77 (0.51, 1.16)	0.214	0.74 (0.50, 1.11)	0.144
	8-12 (n=18)	0.71 (0.41, 1.23)	0.220	0.70 (0.42, 1.17)	0.174
	Days receiving ORT between 6-11 months				
	Base: 0 (n=101)				
	1-7 (n=93)	0.84 (0.65, 1.08)	0.175	0.88 (0.68, 1.13)	0.308
	8-14 (n=27)	0.78 (0.51, 1.19)	0.259	0.79 (0.52, 1.21)	0.281
	15-68 (n=17)	0.77 (0.45, 1.30)	0.324	0.82 (0.48, 1.42)	0.482
Pathogens	Astrovirus (+) at least once (range 0-3 months) between 0-11 months (n=92)	1.18 (0.94, 1.49)	0.145	1.21 (0.96, 1.53)	0.097
	Campylobacter (+) at least once (range 0-9) between 0-5 months (n=191)	1.29 (0.92, 1.81)	0.145	0.27 (0.91, 1.78)	0.153
	Positive E. coli samples associated with diarrhea between 6-11 months				
	Base 0 (n=87)				
	1-3 (n=119)	0.93 (0.73, 1.19)	0.566	0.94 (0.74, 1.20)	0.626
	4-9 (n=32)	0.80 (0.53, 1.20)	0.285	0.81 (0.54, 1.20)	0.294
	Positive E. coli samples associated with diarrhea between 0-11 months				
	Base 0 (n=44)				

	1-3 (n=105)	0.90 (0.68, 1.19)	0.449	0.92 (0.70, 1.21)	0.563
	4-7 (n=70)	0.78 (0.57, 1.09)	0.145	0.81 (0.59, 1.12)	0.213
	8-12 (n=19)	0.66 (0.37, 1.17)	0.158	0.70 (0.40, 1.25)	0.230
Blood, urine, and fecal biomarkers	No variables entered				

Table S2. Univariate models for underweight at 54-66 months (n=239)

Controlling variables	Crude RR (95% CI)	p-value
Girls	1.17 (0.89, 1.53)	0.258
First available weight, kg	0.46 (0.34, 0.61)	<0.0001
Income (rupees x10^3, quantiles)		
Base: 20.1-70 (n=52)		
<8 (n=56)	1.76 (1.13, 2.76)	0.013
8-12.5 (n=67)	1.44 (0.92, 2.26)	0.107
12.6-20 (n=63)	1.35 (0.85, 2.16)	0.206
Constant	2.13 (0.79, 5.62)	0.134

Variable group	Variables entering ≥500 bootstrap repetitions	RR (95% CI)	Unadjusted p-value	RR (95% CI)	Adjusted for gender, first weight and income p-value
Socio-demographic	Sum of assets (0-8)				
	Base: 0 (n=45)				
	1-3 (n=105)	0.82 (0.59, 1.15)	0.254	0.93 (0.67, 1.29)	0.669
	4-8 (n=89)	0.67 (0.46, 0.97)	0.036	0.85 (0.53, 1.36)	0.496
	Food insecurity category				
	Base: no food insecurity (n=68)				
	Mild (n=74)	0.82 (0.54, 1.25)	0.361	0.71 (0.45, 1.11)	0.132
	Moderate-severe (n-97)	1.23 (0.88, 1.72)	0.221	0.96 (0.64, 1.45)	0.867
Breastfeeding	Mean times/d receiving breastfeeding between 6-8 months				
	Base:0-8 (n=12)				
	9-12 (n=66)	0.93 (0.55, 1.58)	0.803	0.91 (0.58, 1.42)	0.685
	13-16 (n=116)	0.69 (0.41, 1.18)	0.176	0.71 (0.46, 1.09)	0.120
	17-21 (n=30)	0.63 (0.32, 1.23)	0.176	0.62 (0.34, 1.14)	0.127
Diet diversity	Started commercial foods after 4 months (n=28)	0.61 (0.34, 1.13)	0.115	0.59 (0.33, 1.04)	0.071
Mean dietary intakes/day 0-8 mo	Received animal-source foods (any quantity/day) between 8-11 months (n=27)	0.55 (0.29, 1.07)	0.078	0.61 (0.33, 1.14)	0.123
	Received ≥1 portion/d of animal-source foods between 8-11 mo (n=12)	0.18 (0.03, 1.19)	0.075	0.20 (0.03, 1.37)	0.103
	Received fruits (any quantity) between 8-11 months (n=92)	0.75 (0.55, 1.03)	0.079	0.81 (0.60, 1.10)	0.181
	Received ≥1 portion/d of fruit between 8-11 months (n=43)	0.64 (0.39, 1.03)	0.065	0.66 (0.42, 1.04)	0.075
	Received 1-2 cups of tea/coffee per day between 8-11 months (n=177)	1.34 (0.92, 1.94)	0.124	1.35 (0.94, 1.95)	0.107
Dietary intakes (yes/no) per period	Received formula between 0-5 months (n=31)	0.62 (0.35, 1.10)	0.103	0.57 (0.34, 0.97)	0.039
	Received formula between 6-11 months (n=13)	1.60 (1.08, 2.38)	0.018	1.45 (0.99, 2.14)	0.057
	Received sweets between 0-5 months (n=163)	0.72 (0.53, 0.98)	0.039	0.77 (0.57, 1.04)	0.089

	Received commercial foods between 6-11 months (n=23)	0.36 (0.15, 0.90)	0.028	0.35 (0.15, 0.84)	0.019
Illness	Mean days/month presenting ALRI between 6-11 months				
	Base: 0 (n=119)				
	1 (n=94)	1.13 (0.83, 1.55)	0.428	1.13 (0.84, 1.52)	0.412
	2 (n=17)	1.46 (0.92, 2.30)	0.104	1.53 (1.00, 2.32)	0.048
	3 (n=9)	1.38 (0.74, 2.57)	0.316	1.30 (0.734, 2.28)	0.359
	Number of ALRI episodes between 6-11 months				
	Base: 0-1 (n=120)				
	2 (n=51)	1.29 (0.91, 1.82)	0.148	1.20 (0.87, 1.66)	0.260
	3 (n=32)	1.34 (0.91, 1.98)	0.138	1.27 (0.90, 1.80)	0.171
	4-6 (n=27)	1.12 (0.70, 1.80)	0.628	1.27 (0.78, 2.08)	0.328
Pathogens	Norovirus (+) at least once between 0-11 months (range: 0-7), n=173	1.5 (0.99, 2.33)	0.051	1.39 (0.93, 2.08)	0.107
	Campylobacter (+) at least once between 0-5 months (range: 0-9), n=192	1.61 (1.01, 2.56)	0.045	1.63 (1.09, 2.44)	0.018
	Enterotoxigenic E. coli (+) at least once between 0-5 months (range=0-3) n=56	0.71 (0.48, 1.06)	0.096	0.72 (0.49, 1.06)	0.098
	Shigella (+) at least once between 6-11 months (range 0-2), n=15	1.55 (1.05, 2.29)	0.026	1.38 (0.93, 2.06)	0.112
Blood, urine and fecal biomarkers	sTfR (mg/L), quantiles				
	Base: 0.2-1.8 mg/L (n=58)				
	1.81-3.0 (n-54)	0.77 (0.52, 1.13)	0.191	0.93 (0.64, 1.33)	0.682
	3.1-4.5 (n=52)	0.91 (0.63, 1.30)	0.590	0.95 (0.68, 1.33)	0.759
	4.6-10.0 (n=52)	0.56 (0.35, 0.89)	0.015	0.54 (0.35, 0.84)	0.006
	Mean neopterin 0-11 months, nmol/L	1.01 (0.97, 1.04)	0.685	1.02 (0.99, 1.05)	0.179
	Neopterin 0-11 months >7 nmol/L (n=23)	1.67 (1.22, 2.28)	0.001	1.83 (1.36, 2.46)	<0.0001

Table S3. Univariate models for underweight + stunting compared with non-stunted children at 54-66 months (n=197)

Controlling variables	Crude RR (95% CI)	p-value
Girls	1.14 (0.85, 1.53)	0.368
First available weight, kg	0.52 (0.37, 0.71)	<0.0001
Income (rupees x10^3, quantiles)		
Base: 20.1-70 (n=52)		
<8 (n=56)	2.07 (1.23, 3.49)	0.006
8-12.5 (n=67)	1.67 (0.99, 2.84)	0.056
12.6-20 (n=63)	1.41 (0.81, 2.46)	0.224
Constant	1.42 (0.47, 4.27)	0.528

Variable group	Variables entering ≥500 bootstrap repetitions	RR (95% CI)	Unadjusted p-value	RR (95% CI)	Adjusted for gender, first weight and income p-value
Socio-demographic	Sum of assets (0-8)				
	Base: 0 (n=39)				
	1-3 (n=86)	0.80 (0.56, 1.15)	0.237	0.91 (0.64, 1.31)	0.622

	4-8 (n=72)	0.71 (0.48, 1.06)	0.095	0.99 (0.60, 1.64)	0.973
	Food insecurity category				
	Base: no food insecurity (n=60)				
	Mild (n=53)	0.95 (0.61, 1.49)	0.826	0.77 (0.47, 1.27)	0.310
	Moderate-severe (n=84)	1.26 (0.87, 1.81)	0.217	0.93 (0.59, 1.48)	0.772
BF	No variables entered				
Diet diversity	Age (months) when started roots				
	Base: ≥6 months (n=60)				
	0-5 months (n=11)	1.40 (0.95, 2.07)	0.086	1.22 (0.84, 1.80)	0.295
	Did not receive roots (n=126)	1.82 (1.03, 3.20)	0.038	1.76 (1.05, 2.96)	0.031
Mean dietary intakes/day 0-8 months	Mean intake of tea/coffee per day between 8-11 months				
	Base: 0 (n=53)				
	1 (n=93)	1.55 (1.02, 2.37)	0.042	1.49 (0.99, 2.24)	0.054
	2 (n=51)	1.33 (0.82, 2.15)	0.250	1.43 (0.89, 2.30)	0.135
	Received tea/coffee between 8-11 months (n=144)	1.47 (0.98, 2.22)	0.065	1.47 (0.99, 2.19)	0.055
	Received ≥1 portion/d of fruit between 8-11 months (n=36)	0.62 (0.37, 1.04)	0.073	0.66 (0.40, 1.09)	0.107
Dietary intakes (yes/no) per period	Receiving dairy products between 0-5 months (n=18)	0.57 (0.26, 1.22)	0.150	0.58 (0.29, 1.17)	0.129
	Receiving commercial foods between 6-11 months (n=19)	0.43 (0.18, 1.04)	0.061	0.46 (0.20, 1.08)	0.075
Illness	Hospitalized between 0-11 months (n=11)	1.65 (1.11, 2.45)	0.014	1.63 (1.15, 2.29)	0.005
	Mean days/month presenting ALRI between 6-11 months				
	Base: 0 (n=98)				
	1 (n=80)	1.10 (0.78, 1.55)	0.575	1.07 (0.80, 1.48)	0.684
	2 (n=13)	1.69 (1.10, 2.62)	0.017	1.63 (1.06, 2.52)	0.027
	3-4 (n=6)	2.04 (1.33, 3.14)	0.001	1.79 (1.19, 2.70)	0.005
	Number of ALRI episodes between 6-11 months				
	Base: 0-1 (n=106)				
	2 (n=45)	1.38 (0.96, 1.98)	0.084	1.27 (0.91, 1.79)	0.163
	3 (n=25)	1.55 (1.04, 2.32)	0.032	1.38 (0.93, 2.04)	0.108
	4 (n=21)	1.23 (0.74,2.05)	0.424	1.31 (0.79, 2.28)	0.291
	Number of diarrheal episodes between 0-11 months				
	Base 0-3 (n=39)				
	4-6 (n=51)	1.19 (0.78, 1.81)	0.420	1.04 (0.69, 1.57)	0.858
	7-9 (n=45)	0.91 (0.56, 1.48)	0.718	0.80 (0.50, 1.28)	0.354
	10-12 (n=33)	1.05 (0.64, 1.71)	0.844	0.93 (0.59, 1.46)	0.753

	13-21 (n=29)	0.67 (0.35, 1.28)	0.225	0.68 (0.37, 1.26)	0.227
	Days receiving ORT between 6-11 months				
	Base: 0 (n=85)				
	1-7 (n=75)	0.74 (0.52, 1.04)	0.082	0.75 (0.55, 1.04)	0.082
	8-14 (n=25)	0.81 (0.50, 1.32)	0.403	0.81 (0.51, 1.29)	0.381
	15-68 (n=12)	0.46 (0.17, 1.26)	0.131	0.54 (0.18, 1.56)	0.252
Pathogens	Campylobacter (+) at least once between 0-5 months (range: 0-9), n=158	1.46 (0.91, 2.35)	0.116	1.46 (0.95, 2.26)	0.086
	Enteroaggregative E. coli (+) at least once between 6-11 months (range: 0-7), n=181	1.90 (0.80, 4.51)	0.145	1.77 (0.77, 4.09)	0.181
	Days of diarrhea associated with E. coli between 0-5 months				
	Base: 0 (n=63)				
	1-3 (n=113)	0.81 (0.59, 1.11)	0.189	0.89 (0.65, 1.21)	0.454
	4-8 (n=21)	0.62 (0.32, 1.19)	0.155	0.38 (0.33, 1.38)	0.286
	Days of diarrhea associated with E. coli between 0-11 months				
	Base: 0 (n=37)				
	1-3 (n=86)	0.84 (0.59, 1.20)	0.341	0.85 (0.60, 1.20)	0.371
	4-7 (n=57)	0.68 (0.44, 1.05)	0.081	0.71 (0.46, 1.08)	0.113
	8-12 (n=17)	0.62 (0.31, 1.26)	0.186	0.72 (0.36, 1.44)	0.356
Blood, urine, and fecal biomarkers	Mean neopterin 0-11 months, nmol/L	1.01 (0.98, 1.04)	0.678	1.02 (0.99, 1.05)	0.185
	Neopterin 0-11 months >7 nmol/L (n=22)	1.46 (1.02, 2.10)	0.037	1.69 (1.22, 2.34)	0.001

Table S4. Univariate models for LAZ at 54-66 months

Controlling variables	Coeff ± SE 95% CI	p-value
Girls	-0.21 ± 0.12<br>-0.45, 0.02	0.071
First available weight, kg	0.46 ± 0.12<br>0.23, 0.69	<0.0001
Income (rupees x10^3, quantiles)		
Base: 20.1-70 (n=52)		
<8 (n=56)	-0.47 ± 0.17<br>-0.81, -0.12	0.008
8-12.5 (n=67)	-0.36 ± 0.17<br>-0.69, -0.03	0.032
12.6-20 (n=63)	-0.21 ± 0.17<br>-0.54, 0.12	0.218
Constant	-2.83 ± 0.41<br>-3.64, -2.01	<0.0001

Variable group	Variables entering ≥ 500 bootstrap repetitions	Unadjusted	Adjusted for gender, income and first weight

		Coeff ± SE 95% CI	p-value	Coeff ± SE 95% CI	p-value
Socio-demographic	Sum of assets (range: 0-8)				
	Base: 0 (n=44)				
	1-3 (n=105)	0.13 ± 0.17<br>-0.20, 0.46	0.434	0.03 ± 0.16<br>-0.30, 0.35	0.873
	4-8 (n=89)	0.29 ± 0.17<br>-0.255, 0.63	0.099	0.07 ± 0.19<br>-0.31, 0.45	0.710
	Food insecurity category				
	Base: no food insecurity (n=68)				
	Mild (n=74)	-0.14 ± 0.16<br>-0.45, 0.16	0.360	-0.07 ± 0.16<br>-0.38, 0.24	0.675
	Moderate-severe (n-96)	-0.29 ± 0.15<br>-0.58, 0.006	0.055	-0.14 ± 0.17<br>-0.47, 0.19	0.398
Breastfeeding	Mean times/d receiving formula between 0-5 months				
	Base: 0 (n=207)				
	1-2 (n=21)	0.26 ± 0.21<br>-0.16, 0.68	0.220	0.34 ± 0.20<br>-0.07, 0.74	0.103
	3-10 (n=10)	0.83 ± 0.30<br>0.23, 1.42	0.006	0.77 ± 0.30<br>0.17, 1.36	0.012
Diet diversity	Age starting formula				
	Base: did not receive formula (n=203)				
	<1 month (n=10)	0.46 ± 0.30<br>-0.14, 1.05	0.133	0.33 ± 0.29<br>-0.25, 0.91	0.270
	1 month (n=12)	0.41 ± 0.28<br>-0.14, 0.96	0.143	0.37 ± 0.28<br>-0.17, 0.92	0.179
	2-6 months (n=13)	0.04 ± 0.27<br>-0.48, 0.57	0.868	0.20 ± 0.26<br>-0.31, 0.72	0.439
	Age starting roots				
	Base: did not receive roots at 8 months (n=155)				
	1-5 months (n=14)	-0.04 ± 0.26<br>-0.55, 0.47	0.885	-0.08 ± 0.25<br>-0.58, 0.41	0.734
	6-8 months (n=69)	0.29 ± 0.13<br>0.03, 0.56	0.030	0.23 ± 0.13<br>-0.03, 0.49	0.080
	Times/d being fed between 6-8 months				
	Base: 0 (n=204)				
	1-2 (n=15)	-0.17 ± 0.25<br>-0.67, 0.32	0.484	-0.13 ± 0.24<br>-0.60, 0.35	0.595
	3-4 (n=12)	-0.47 ± 0.28<br>-1.02, 0.07	0.091	-0.35 ± 0.27<br>-0.88, 0.17	0.189
Mean dietary intakes/day 0-8 months	Received >=1 portion/d of protein between 8-11 months (n=12)	0.76 ± 0.27<br>0.22, 1.31	0.006	0.71 ± 0.26<br>0.19, 1.24	0.007<br>Excluded due to heteroscedasticity
Dietary intakes (yes/no) per period	Received formula between 0-5 months (n=31)	0.44 ± 0.18<br>0.09, 0.80	0.014	0.46 ± 0.18<br>0.11, 0.81	0.010
	Received dairy products between 0-5 months (n=19)	0.48 ± 0.22<br>0.04, 0.92	0.033	0.51 ± 0.21<br>0.09, 0.93	0.018
	Received roots between 6-11 months (n=80)	0.26 ± 0.13<br>0.007, 0.51	0.044	0.20 ± 0.13<br>-0.05, 0.45	0.118
	Received commercial foods between 6-11 months (n=23)	0.51 ± 0.20<br>0.11, 0.91	0.012	0.56 ± 0.20<br>0.18, 0.95	0.004
Illness	Hospitalized at least once between 0-11 months (range 0-8), n=13	-0.64 ± 0.26<br>-1.16, -0.11	0.017	-0.58 ± 0.25<br>-1.08, -0.08	0.024
	Days presenting ear pain/pulling between 0-11 months				
	Base: 0 (n=40)				
	1-14 (n=77)	0.07 ± 0.18<br>-0.29, 0.44	0.687	0.04 ± 0.17<br>-0.30, 0.39	0.800
	15-28 (n=36)	0.08 ± 0.22<br>-0.34, 0.51	0.693	0.18 ± 0.21<br>-0.23, 0.59	0.380

29-312 (n=85)	-0.05 ± 0.18<br>-0.41, 0.30	0.773	-0.02 ± 0.17<br>-0.36, 0.32	0.900
Mean days/month presenting vomit between 0-11 months				
Base: 0 (n=60)				
1-3 (n=100)	-0.40 ± 0.15<br>-0.70, -0.10	0.009	-0.44 ± 0.14<br>-0.72, -0.15	0.003
4-14 (n=40)	-0.24 ± 0.19<br>-0.62, 0.13	0.197	-0.23 ± 0.18<br>-0.58, 0.13	0.211
15-30 (n=38)	-0.49 ± 0.19<br>-0.87, -0.11	0.011	-0.44 ± 0.18<br>-0.80, -0.08	0.016
Mean days/month presenting vomit between 0-5 months				
Base: 0 (n=80)				
1-3 (n=83)	-0.27 ± 0.15<br>-0.56, 0.02	0.069	-0.32 ± 0.14<br>-0.60, -0.04	0.023
4-14 (n=29)	-0.23 ± 0.20<br>-0.63, 0.16	0.250	-0.27 ± 0.19<br>-0.65, 0.11	0.164
15-30 (n=46)	-0.33 ± 0.17<br>-0.67, 0.01	0.057	-0.26 ± 0.16<br>-0.59, 0.06	0.112
Mean days/month presenting vomit between 6-11 months				
Base: 0 (n=60)				
1-3 (n=112)	-0.04 ± 0.15<br>0.034, 0.25	0.759	-0.05 ± 0.14<br>-0.34, 0.23	0.699
4-14 (n=38)	0.06 ± 0.19<br>-0.32, 0.44	0.766	0.10 ± 0.18<br>-0.27, 0.46	0.595
15-30 (n=28)	-0.47 ± 0.21<br>-0.89, -0.05	0.028	-0,42 ± 0.20<br>-0.82, -0.02	0.040
Mean days/month presenting ALRI between 0-11 months				
Base: 0 (n=119)				
1 (n=94)	-0.16 ± 0.13<br>-0.42, 0.10	0.235	-0.14 ± 0.13<br>-0.39, 0.11	0.266
2 (n=16)	-0.44 ± 0.20<br>-0.84, -0.05	0.028	-0.50 ± 0.19<br>-0.87, -0.12	0.010
3-4 (n=9)	-0.22 ± 0.31<br>-0.83, 0.39	0.477	-0.23 ± 0.30<br>-0.83, 0.37	0.444
Mean days/month presenting ALRI between 6-11 months				
Base: 0 (n=133)				
1 (n=100)	-0.12 ± 0.13<br>-0.37, 0.13	0.336	-0.14 ± 0.12<br>-0.39, 0.10	0.238
2 (n=21)	-0.44 ± 0.25<br>-0.93, 0.04	0.075	-0.53 ± 0.23<br>-1.00, -0.07	0.024
3-4 (n=11)	-0.80 ± 0.32<br>-1.44, -0.17	0.013	-0.82 ± 0.31<br>-1.43, -0.20	0.009
Number of ALRI episodes between 0-11 months				[non-significant in the composite model]
Base: 0-1 (n=47)				
2-4 (n=124)	-0.29 ± 0.16<br>-0.61, 0.02	0.068	-0.35 ± 0.15<br>-0.65, -0.05	0.022
5-10 (n=67)	-0.40 ± 0.18<br>-0.75, -0.05	0.025	-0.49 ± 0.17<br>-0.83, -0.15	0.005
Number of ALRI episodes between 6-11 months				
Base: 0				
1 (n=86)	0.07 ± 0.16<br>-0.24, 0.39	0.653	-0.17 ± 0.17<br>-0.50, 0.16	0.305
2 (n=51)	0.07 ± 0.17<br>-0.27, 0.41	0.685	-0.42 ± 0.18<br>-0.79, -0.05	0.024
3 (n=31)	0.23 ± 0.20<br>-0.17, 0.64	0.255	-0.45 ± 0.21<br>-0.86, -0.04	0.033

	4-6 (n=27)	0.40 ± 0.25<br>-0.09, 0.89	0.111	-0.45 ± 0.22<br>-0.89, -0.02	0.042
	Number of diarrheal episodes between 6-11 months				
	Base: 0-1 (n=67)				
	2-3 (n=70)	0.07 ± 0.16<br>-0.24, 0.39	0.653	0.08 ± 0.15<br>-0.22, 0.38	0.612
	4-5 (n=52)	0.07 ± 0.17<br>-0.27, 0.41	0.685	0.06 ± 0.17<br>-0.27, 0.39	0.735
	6-7 (n=31)	0.23 ± 0.20<br>-0.17, 0.64	0.255	0.26 ± 0.19<br>-0.12, 0.65	0.177
	8-12 (n=18)	0.40 ± 0.25<br>-0.09, 0.89	0.111	0.40 ± 0.24<br>-0.08, 0.87	0.100
	Days receiving ORT between 6-11 months				
	Base: 0 (n=101)				
	1-7 (n=93)	0.31 ± 0.13<br>0.05, 0.58	0.020	0.24 ± 0.13<br>-0.02, 0.49	0.074
	8-14 (n=27)	0.11 ± 0.20<br>-0.28, 0.51	0.568	0.11 ± 0.19<br>-0.27, 0.50	0.565
	15-68 (n=17)	0.37 ± 0.24<br>-0.11, 0.85	0.129	0.26 ± 0.24<br>-0.21, 0.72	0.282
Pathogens	Rotavirus (+) at least once between 0-5 months (range: 0-2), n=32	-0.30 ± 0.18<br>-0.65, 0.05	0.090	-0.24 ± 0.17<br>-0.57, 0.10	0.161
	Days of diarrhea associated with Enteroaggregative E. coli between 6-11 months				
	Base: 0 (n=152)				
	1 (n=55)	0.61 ± 0.15	0.278	0.15 ± 0.14<br>-0.13, 0.42	0.305
	2-5 (n=31)	0.31 ± 0.18<br>-0.05, 0.67	0.092	0.30 ± 0.18<br>-0.05, 0.66	0.094
	Days of diarrhea associated with Enteroaggregative E. coli between 0-11 months				+ Adjusting for severity of diarrhea by EAEC
	Base: 0 (n=105)				
	1 (n=53)	0.03 ± 0.16<br>-0.28, 0.34	0.861	-0.11 ± 0.25<br>-0.62, 0.39	0.652
	2 (n=44)	-0.04 ± 0.17<br>-0.37, 0.29	0.815	-0.17 ± 0.25<br>-0.67, 0.32	0.489
	3-7 (n=36)	0.40 ± 0.18<br>0.05, 0.76	0.026	0.29 ± 0.26<br>-0.22, 0.81	0.260
Blood, urine and fecal biomarkers	Mean alpha-1 antitrypsin, mg/g between 0-5 months	-0.01 ± 0.005<br>-0.02, 0.001	0.078	-0.01 ± 0.005<br>-0.02, 0.004	0.226
	Mean alpha-1 antitrypsin, mg/g between 6-11 months	-0.01 ± 0.005<br>-0.02, 0.001	0.078	-0.006 ± 0.005<br>-0.01, 0.003	0.207
	Mean alpha-1 antitrypsin, mg/g between 0-11 months	-0.01 ± 0.005<br>-0.02, 0.001	0.071	-0.006 ± 0.005<br>-0.01, 0.003	0.215
	Mean alpha-1 antitrypsin >20 mg/g between 0-11 months (visual point of change in distribution, n=29)	-0.37 ± 0.19<br>-0.76, 0.01	0.056	-0.27 ± 0.19<br>-0.63, 0.10	0.155
	Mean neopterin nmol/L between 0-5 months	-0.04 ± 0.02<br>-0.08, 0.01	0.121	-0.03 ± 0.02<br>-0.08, 0.01	0.124
	Mean neopterin >7 nmol/L between 0-5 months	-0.22 ± 0.21<br>-0.64, 0.20	0.306	-0.28 ± 0.20<br>-0.69, 0.12	0.171
	Mean neopterin, nmol/L between 6-11 months	-0.03 ± 0.02<br>-0.08, 0.01	0.105	-0.03 ± 0.02<br>-0.07, 0.01	0.127
	Mean neopterin >7 nmol/L between 6-11 months	-0.26 ± 0.21<br>-0.68, 1.16	0.223	-0.31 ± 0.20<br>-0.71, 0.09	0.130

Table S5. Univariate models for WAZ at 54-66 months

Controlling variables	Coeff ± SE<br>95% CI	p-value
Girls	-0.18 ± 0.11<br>-0.40, 0.04	0.109
First available weight, kg	0.57 ± 0.11<br>0.35, 0.79	<0.0001
Income (rupees x10^3, quantiles)		
Base: 20.1-70 (n=52)		
<8 (n=56)	-0.29 ± 0.16<br>-0.62, 0.03	0.076
8-12.5 (n=67)	-0.25 ± 0.16<br>-0.56, 0.06	0.114
12.6-20 (n=63)	-0.10 ± 0.16<br>-0.42, 0.21	0.510
Constant	-3.10 ± 0.39<br>-3.87, -2.33	<0.0001

Variable group	Variables entering ≥ 500 bootstrap repetitions	Coeff ± SE<br>95% CI	Unadjusted p-value	Coeff ± SE<br>95% CI	Adjusted for gender, income and first weight p-value
Socio-demographic	Number of assets				
	Base: 0 (n=45)				
	0-3 (n=105)	0.03 ± 0.16<br>-0.28, 0.35	0.840	-0.07 ± 0.15<br>-0.37, 0.23	0.657
	4-8 (n=89)	0.21 ± 0.16<br>-0.12, 0.53	0.211	0.02 ± 0.18<br>-0.34, 0.37	0.928
Breastfeeding	Minimal age starting predominant breastfeeding				
	Base: <1 month (n=165)				
	In the first month (n=22)	-0.20 ± 0.20<br>-0.60, 0.21	0.339	-0.30 ± 0.19<br>-0.68, 0.08	0.117
	Between 2-6 months (n=15)	-0.38 ± 0.24<br>-0.86, 0.10	0.117	-0.27 ± 0.23<br>-0.72, 0.19	0.246
	Minimal age starting formula				
	Base: did not receive formula (n=204)				
	<1 month (n=10)	0.69 ± 0.29<br>0.12, 1.26	0.018	0.57 ± 0.28<br>0.03, 1.12	0.040
	In the first month (n=12)	0.08 ± 0.26<br>-0.44, 0.61	0.747	0.08 ± 0.26<br>-0.43, 0.60	0.742
	2-6 months (n=13)	-0.16 ± 0.25<br>-0.66, 0.35	0.540	0.03 ± 0.24<br>-0.45, 0.52	0.889
	Mean times/d receiving breastfeeding between 6-8 months				
	Base: 0-8 (n=12)				
	9-12 (n=66)	0-0.08 ± 0.27<br>-0.62, 0.46	0.760	-0.04 ± 0.26<br>-0.55, 0.46	0.860
	13-16 (n=116)	0.22 ± 0.26<br>-0.30, 0.74	0.413	0.22 ± 0.25<br>-0.26, 0.71	0.370
	17-21 (n=30)	0.13 ± 0.30<br>-0.45, 0.72	0.654	0.14 ± 0.28<br>-0.41, 0.69	0.612
Diet diversity	Started commercial foods after 4 months (n=28)	0.35 ± 0.18<br>-0.002, 0.71	0.051	0.44 ± 0.17<br>0.11, 0.77	0.010

Mean dietary intakes/day 0-8 months	Mean frequency of breastfeeding /day between 6-8 months<br>Base: 5-12 (n=50)				
	13-18 (n=154)	0.26 ± 0.14<br>-0.02, 0.55	0.065	0.22 ± 0.13<br>-0.04, 0.48	0.104
	19-25 (n=20)	0.15 ± 0.23<br>-0.30, 0.61	0.504	0.16 ± 0.22<br>-0.27, 0.59	0.465
	Mean cups/d of tea/coffee taken between 8-11 months<br>Base: 0 (n=62)				
	1 cup (n=113)	-0.29 ± 0.14<br>-0.57, -0.012	0.041	-0.25 ± 0.13<br>-0.51, 0.01	0.063
	2 cups (n=64)	-0.20 ± 0.16<br>-0.52, 0.11	0.200	-0.21 ± 0.15<br>-0.50, 0.09	0.170
	Taking a mean of 1 cup of tea/coffee per day between 8-11 months (n= 177)	-0.26 ± 0.13<br>-0.52, 0.0004	0.050	-0.23 ± 0.12<br>-0.48, 0.01	0.061
	Taking a mean of 1 portion of animal-source foods per day between 8-11 months (n=12)	0.68 ± 0.26<br>0.17, 1.20	0.010	0.64 ± 0.25<br>0.15, 1.13	0.011
	Received any quantity of animal-source foods between 8-11 months (n=27)	0.39 ± 0.18<br>0.03, 0.75	0.035	0.33 ± 0.17<br>-0.01, 0.67	0.058
	Received a mean of 1 portion of fruit/day between 8-11 months (n=43)	0.31 ± 0.15<br>0.02, 0.61	0.037	0.31 ± 0.14<br>0.03, 0.58	0.030
	Received any quantity of fruits between 8-11 months (n=92)	0.19 ± 0.12<br>-0.05, 0.42	0.115	0.15 ± 0.11<br>-0.07, 0.37	0.190
	Receiving sweets between 0-5 months (n=163)	0.28 ± 0.15<br>-0.02, 0.58	0.064	0.24 ± 0.14<br>-0.03, 0.52	0.087
	Receiving commercial foods between 6-11 months (n=23)	0.60 ± 0.19<br>0.22, 0.98	0.002	0.67 ± 0.18<br>0.32, 1.03	<0.0001
Illness	Hospitalized at least once between 0-11 months (range: 0-8), n=13	-0.41 ± 0.25<br>-1.02, -0.01	0.045	-0.42 ± 0.24<br>-0.90, 0.05	0.079
	Number of diarrheal episodes between 0-11 months<br>Base 0-3 (n=46)				
	4-6 (n=63)	-0.13 ± 0.17<br>-0.48, 0.21	0.446	-0.05 ± 0.16<br>-0.37, 0.28	0.774
	7-9 (n=53)	0.07 ± 0.18<br>-0.40, 0.36	0.694	0.12 ± 0.17<br>-0.22, 0.46	0.490
	10-12 (n=41)	-0.02 ± 0.19<br>-0.40, 0.36	0.924	0.03 ± 0.18<br>-0.33 ± 0.39	0.879
	13-21 (n=36)	0.25 ± 0.20<br>-0.15, 0.64	0.220	0.19 ± 0.19<br>-0.18, 0.57	0.312
	Number of diarrheal episodes between 6-11 months<br>Base: 0-1 (n=67)				+ Adjusting for severity of diarrhea 6-11 months
	2-3 (n=71)	0.10 ± 0.15<br>-0.20, 0.40	0.502	0.01 ± 0.16<br>-0.30, 0.32	0.934
	4-5 (n=52)	0.19 ± 0.16<br>-0.14, 0.51	0.254	0.09 ± 0.17<br>-0.25, 0.43	0.615
	5-7 (n=31)	0.41 ± 0.19<br>0.03, 0.79	0.036	0.34 ± 0.19<br>-0.05, 0.72	0.085
	8-12 (n=18)	0.38 ± 0.24<br>-0.09, 0.85	0.110	0.28 ± 0.24<br>-0.18, 0.75	0.235
	Mean days/month presenting low appetite between 0-5 months<br>Base: 0 (n=175)				
	1 (n=49)	-0.28 ± 0.14<br>-0.57, -0.003	0.048	-0.25 ± 0.13<br>-0.51, 0.01	0.064
	2-8 (n=15)	-0.56 ± 0.24	0.019	-0.49 ± 0.22	0.031

		-1.03, -0.09		-0.93, -0.04	
	Mean days/month presenting ALRI between 6-11 months				
	Base: 0 (n=119)				
	1 (n=94)	-0.07 ± 0.12<br>-0.31, 0.18	0.595	-0.08 ± 0.12<br>-0.31, 0.15	0.473
	2 (n=17)	-0.09 ± 0.23<br>-0.55, 0.37	0.698	-0.17 ± 0.22<br>-0.60, 0.27	0.448
	3-4 (n=9)	-0.29 ± 0.31<br>-0.91, 0.32	0.346	-0.28 ± 0.30<br>-0.87, 0.31	0.352
	Number of ALRI episodes between 6-11 months				
	Base: 0-1 (n=129)				
	2 (n=51)	-0.20 ± 0.15<br>-0.50, 0.09	0.173	-0.17 ± 0.14<br>-0.44, 0.11	0.235
	3 (n=32)	-0.11 ± 0.18<br>-0.46, 0.24	0.530	-0.11 ± 0.17<br>-0.44, 0.21	0.488
	4-6 (n=27)	-0.15 ± 0.19<br>-0.53, 0.22	0.420	-0.27 ± 0.18<br>-0.63, 0.09	0.137
Pathogens	Norovirus (+) between 0-11 months (range: 0-7), n=173	-0.26 ± 0.14<br>-0.54, 0.01	0.063	-0.19 ± 0.13<br>-0.45, 0.07	0.152
	Norovirus (+) between 0-5 months (range: 0-4), n=122	-0.22 ± 0.11<br>-0.45, 0.008	0.058	-0.19 ± 0.11<br>-0.41, 0.02	0.078
	Days of diarrhea associated with E. coli between 0-5 months				
	Base 0 (n=74)				
	1-3 (n=139)	0.26 ± 0.13<br>0.004, 0.51	0.046	0.16 ± 0.12<br>-0.08, 0.40	0.188
	4-8 (n=26)	0.38 ± 0.20<br>-0.02, 0.78	0.066	0.28 ± 0.19<br>-0.10, 0.66	0.143
	Positive E.coli samples associated with diarrhea between 6-11 months				
	Base: 0 (n=88)				
	1-3 (n=119)	0.15 ± 0.13<br>-0.10, 0.40	0.240	0.12 ± 0.12<br>-0.12, 0.36	0.339
	4-9 (n=32)	0.32 ± 0.18<br>-0.05, 0.68	0.088	0.27 ± 0.39<br>-0.07, 0.61	0.125
	Positive E. coli samples associated with diarrhea between 0-11 months				
	Base: 0 (n=45)				
	1-3 (n=105)	0.23 ± 0.16<br>-0.08, 0.55	0.146	0.18 ± 0.15<br>-0.12, 0.47	0.243
	4-7 (n=70)	0.28 ± 0.17<br>-0.05, 0.62	0.097	0.19 ± 0.16<br>-0.13, 0.51	0.245
	8-12 (n=19)	0.46 ± 0.24<br>-0.02, 0.95	0.059	0.30 ± 0.23<br>-0.15, 0.76	0.194
	Positive Enteroaggregative E. coli samples associated with diarrhea between 6-11 months				
	Base: 0 (n=153)				
	1 (n=55)	0.22 ± 0.14<br>-0.05, 0.50	0.115	0.20 ± 0.13<br>-0.06, 0.46	0.138
	2-5 (n=31)	0.23 ± 0.18<br>-0.11, 0.58	0.188	0.20 ± 0.38<br>-0.14, 0.53	0.248
	Positive Enteroaggregative E. coli samples associated with diarrhea between 0-11 months				+Adjusting for severity of diarrhea associated with EAEC 0-11 months
	Base: 0 (n=106)				
	1 (n=53)	0.19 ± 0.15<br>-0.11, 0.48	0.214	-0.14 ± 0.24<br>-0.61, 0.32	0.548
	2 (n=44)	0.10 ± 0.16	0.547	-0.22 ± 0.24	0.342

		-0.22, 0.41		-0.69, 0.24	
	3-7 (n=36)	0.40 ± 0.17<br>0.06, 0.74	0.020	0.11 ± 0.24<br>-0.37, 0.59	0.657
Blood, urine and fecal biomarkers	sTfR (mg/L), quantiles				
	Base: 0.2-1.8 mg/L (n=58)				
	1.81-3.0 (n-54)	0.31 ± 0.17<br>-0.02, 0.64	0.065	0.18 ± 0.16<br>-0.14, 0.49	0.267
	3.1-4.5 (n=52)	0.22 ± 0.17<br>-0.12, 0.55	0.200	0.19 ± 0.16<br>-0.12, 0.50	0.237
	4.6-10.0 (n=52)	0.58 ± 0.17<br>0.25, 0.92	0.001	0.59 ± 0.16<br>0.28, 0.91	<0.0001

Table S6. Univariate models for WLZ at 54-66 months

**Controlling variables**	**Coeff ± SE**<br>**95% CI**	**p-value**
Girls	0.004 ± 0.11<br>-0.21, 0.21	0.970
First available weight, kg	0.39 ± 0.11<br>0.18, 0.60	<0.0001
Income (rupees x10^3, quantiles)		
Base: 20.1-70 (n=52)		
<8 (n=56)	0.01 ± 0.16<br>-0.30, 0.32	0.935
8-12.5 (n=67)	0.08 ± 0.15<br>-0.22, 0.38	0.608
12.6-20 (n=63)	0.03 ± 0.15<br>-0.27, 0.34	0.821
Constant	-1.88 ± 0.37<br>-2.62, -1.14	<0.0001

**Variable group**	**Variables entering ≥ 500 bootstrap repetitions**	**Coeff ± SE**<br>**95% CI**	**Unadjusted p-value**	**Coeff ± SE**<br>**95% CI**	**Adjusted for gender, income and first weight p-value**
Socio-demographic	No variables entered				
Breastfeeding	Age starting solid/semisolid foods				
	Base: 6-8 months (n=17)				
	0-3 months (n=103)	-0.34 ± 0.22<br>-0.76, 0.09	0.120	-0.26 ± 0.21<br>-0.68, 0.16	0.222
	4-5 months (n=78)	-0.32 ± 0.22<br>-0.75, 0.12	0.152	-0.27 ± 0.22<br>-0.68, 0.16	0.224
	Did not start up to 8 months (n=39)	-0.50 ± 0.24<br>-0.98, -0.03	0.037	-0.45 ± 0.24<br>-0.91, 0.02	0.060
	Age starting milk				
	Base: 6-20 months (n=28)				
	<1 month (n=117)	-0.30 ± 0.17<br>-0.64, 0.04	0.082	-0.28 ± 0.17<br>-0.61, 0.05	0.094
	1-2 months (n=58)	-0.47 ± 0.19<br>-0.84, -0.10	0.012	-0.45 ± 0.18<br>-0.81, -0.09	0.015
	3-5 months (n=30)	-0.02 ± 0.21<br>-044, 0.40	0.921	0.05 ± 0.21<br>-0.36, 0.46	0.802
Diet diversity	**>= 500 reps**				
	Age starting formula				
	Base: did not receive formula (n=202)				
	<1 month (n=10)	0.56 ± 0.27<br>0.03, 1.08	0.037	0.52 ± 0.26<br>-0.006, 1.04	0.053

	1 month (n=12)	-0.29 ± 0.24<br>-0.77, 0.18	0.228	-0.24 ± 0.25<br>-0.73, 0.25	0.338
	2-6 months (n=13)	-0.33 ± 0.23<br>-0.80, 0.13	0.154	-0.20 ± 0.23<br>-0.66, 0.26	0.392
	Age starting legumes				
	Base: 3-11 months (n=26)				
	12-17 months (n=46)	0.41 ± 0.20<br>0.01, 0.81	0.044	0.58 ± 0.20<br>0.18, 0.99	0.005
	18-23 months (n=59)	0.20 ± 0.19<br>-0.18, 0.58	0.307	0.33 ± 0.19<br>-0.05, 0.72	0.086
	24-60 months (n=73)	0.14 ± 0.19<br>-0.23, 0.51	0.458	0.27 ± 0.19<br>-0.10, 0.63	0.156
Mean dietary intakes/day 0-8 months	**>= 500 reps**				
	Taking ≥1 portion/d of animal-source foods between 8-11 months (n=12)	0.26 ± 0.24<br>-0.22, 0.74	0.289	0.23 ± 0.24<br>-0.24, 0.71	0.334
	Received any quantity of animal-source foods/d between 8-11 months (n=27)	0.37 ± 0.17<br>0.04, 0.70	0.030	0.35 ± 0.17<br>0.02, 0.67	0.039
	Receiving ≥1 portion/d of fruits between 8-11 months (n=45)	0.24 ± 0.14<br>-0.03, 0.52	0.081	0.23 ± 0.14<br>-0.03, 0.50	0.085
Dietary intakes (yes/no) per period	Received solids/semisolids between 0-5 months (n=206)	-0.29 ± 0.16<br>-0.60, 0.02	0.071	-0.25 ± 0.16<br>-0.56, 0.06	0.109
	Received sweets between 0—5 months (n=162)	0.30 ± 0.14<br>0.02, 0.58	0.038	0.27 ± 0.14<br>-0.006, 0.54	0.055
	Received sweets between 6-11 months (n=217)	0.37 ± 0.23<br>-0.09, 0.83	0.119	0.30 ± 0.23<br>-0.15, 0.75	0.195
	Received other fluids between 6-11 months (n=170)	0.32 ± 0.12<br>0.09, 0.55	0.008	0.32 ± 0.12<br>0.09, 0.55	0.007
	Received commercial foods between 6-11 months (n=22)	0.34 ± 0.18<br>-0.02, 0.70	0.063	0.40 ± 0.18<br>0.05, 0.76	0.027
Illness	**>= 500 reps**				
	Mean days/month presenting low appetite between 0-5 months				
	Base: 0 (n=174)				
	1 (n=49)	-0.32 ± 0.13<br>-0.58, -0.05	0.018	-0.30 ± 0.13<br>-0.55, -0.04	0.022
	2-8 (n=14)	-0,42 ± 0.23<br>-0.86, 0.03	0.069	-0.37 ± 0.22<br>-0.81, 0.07	0.102
	Mean days/month presenting ALRI between 0-11 months				
	Base: 0 (n=110)				
	1 (n=89)	0.16 ± 0.12<br>-0.07, 0.39	0.168	0.19 ± 0.11<br>-0.03, 0.42	0.090
	2 (n=28)	0.33 ± 0.17<br>-0.01, 0.67	0.061	0.33 ± 0.17<br>-0.01, 0.66	0.059
	3-4 (n=10)	0.29 ± 0.27<br>-0.24, 0.83	0.280	0.38 ± 0.28<br>-0.17, 0.92	0.173
	Mean days/month presenting ALRI between 0-5 months<br>Base: 0 (n=92)				Adjusting for hospitalizations associated with ALRI between -0-5 months
	1 (n=85)	0.20 ± 0.12<br>-0.04, 0.45	0.097	0.23 ± 0.14<br>-0.05, 0.51	0.109
	2 (n=41)	0.46 ± 0.15<br>0.15, 0.76	0.003	0.48 ± 0.17<br>0.15, 0.81	0.005
	3-4 (n=19)	0.26 ± 0.21<br>-0.15, 0.66	0.214	0.22 ± 0.23<br>-0.22, 0.67	0.328

	Number of diarrheal episodes between 6-11 months<br>Base: 0-1 (n=67)				+Adjusting of episode's severity 6-11 months
	2-3 (n=71)	0.15 ± 0.14<br>-0.12, 0.43	0.279	0.11 ± 0.15<br>-0.19, 0.42	0.461
	4-5 (n=51)	0.27 ± 0.15<br>-0.03, 0.57	0.083	0.21 ± 0.17<br>-0.12, 0.55	0.212
	5-7 (n=31)	0.40 ± 0.18<br>0.04, 0.75	0.027	0.35 ± 0.19<br>-0.03, 0.73	0.071
	8-12 (n=17)	0.14 ± 0.22<br>-0.30, 0.58	0.534	0.07 ± 0.24<br>-0.40, 0.54	0.783
Pathogens	Norovirus (+) at least once between 0-11 months (range: 0-7), n=171	-0.29 ± 0.13<br>-0.55, -0.04	0.023	-0.07 ± 0.13<br>-0.52, -0.02	0.036
	Aeromonas (+) at least once between 6-11 months (range: 0-6), n=105	-0.21 ± 0.11<br>-0.42, 0.003	0.053	-0.20 ± 0.11<br>-0.41, 0.01	0.065
Blood, urine and fecal biomarkers	sTfR (mg/L), quantiles<br>Base: 0.2-1.8 mg/L (n=57)				
	1.81-3.0 (n-54)	0.33 ± 0.15<br>0.04, 0.63	0.028	0.27 ± 0.15<br>-0.02, 0.56	0.067
	3.1-4.5 (n=51)	-0.0006 ± 0.15<br>-0.30, 0.30	0.997	0.004 ± 0.15<br>-0.29, 0.29	0.980
	4.6-10.0 (n=52)	0.55 ± 0.15<br>0.25, 0.85	<0.0001	0.60 ± 0.15<br>0.31, 0.90	<0.0001
